# Forecasting of potential anti-inflammatory targets of some immunomodulatory plants and their constituents using *in vitro*, molecular docking and network pharmacology-based analysis

**DOI:** 10.1038/s41598-023-36540-3

**Published:** 2023-06-12

**Authors:** Asmaa Khairy, Doaa A. Ghareeb, Ismail Celik, Hala M. Hammoda, Hala H. Zaatout, Reham S. Ibrahim

**Affiliations:** 1 Department of Pharmacognosy, Faculty of Pharmacy, Alexandria University, Alexandria, 21521 Egypt; 2 Bio-Screening and Preclinical Trial Lab, Biochemistry Department, Faculty of Science, Alexandria University, Alexandria, Egypt; 3 Department of Pharmaceutical Chemistry, Faculty of Pharmacy, Erciyes University, Kayseri, 38039 Turkey

**Keywords:** Drug discovery, Plant sciences

## Abstract

Most synthetic immunomodulatory medications are extremely expensive, have many disadvantages and suffer from a lot of side effects. So that, introducing immunomodulatory reagents from natural sources will have great impact on drug discovery. Therefore, this study aimed to comprehend the mechanism of the immunomodulatory activity of some natural plants via network pharmacology together with molecular docking and *in vitro* testing. Apigenin, luteolin, diallyl trisulfide, silibinin and allicin had the highest percentage of C-T interactions while, AKT1, CASP3, PTGS2, NOS3, TP53 and MMP9 were found to be the most enriched genes. Moreover, the most enriched pathways were pathways in cancer, fluid shear stress and atherosclerosis, relaxin signaling pathway, IL-17 signaling pathway and FoxO signaling pathway. Additionally, *Curcuma longa*, *Allium sativum*, *Oleu europea, Salvia officinalis*, *Glycyrrhiza glabra* and *Silybum marianum* had the highest number of P-C-T-P interactions. Furthermore, molecular docking analysis of the top hit compounds against the most enriched genes revealed that silibinin had the most stabilized interactions with AKT1, CASP3 and TP53, whereas luteolin and apigenin exhibited the most stabilized interactions with AKT1, PTGS2 and TP53. *In vitro* anti-inflammatory and cytotoxicity testing of the highest scoring plants exhibited equivalent outcomes to those of piroxicam.

## Introduction

The human immune system plays a vital role to protect the body from totally different attacks of any morbific microorganisms. Immune system is classified into two categories which include humoral or antibody mediated immunity and cellular mediated immunity^1^. The disorder known as autoimmune illness occurs when the immune system unintentionally targets its own body cells. If the immune system's error is not fixed, the attack may spread to the heart, lungs, and other crucial organs. Uncertainties surround the mechanisms behind immune response dysregulation. It is conceivable that excessive exposure to heavy metals, pathogenic bacteria and parasites, leaky gut syndrome and dietary imbalances can tax the immune system to the point where it becomes dysregulated^2^. Despite the fact that there are more than 100 different forms of autoimmune diseases; rheumatoid arthritis, multiple sclerosis, lupus, psoriasis, type 1 diabetes and others are the most prevalent ones^3^. The global autoimmune disease therapeutics cost is very huge, where the overall cost of autoimmune diseases therapeutics worldwide in 2021 was evaluated by 127 billion dollars and is projected to reach 149 billion dollars by 2023^4^. Medication associated with autoimmune disorders treatment is one of the most multifaceted and challenging areas of modern medicine. This evokes pharmaceutical companies for the continued research for new drug candidates in this area. Different varieties of drugs including NSAIDs, anti-inflammatories, immune suppressants, corticosteroids, interferons and others, are approved for autoimmune disorders treatment. For example, the anti-inflammatory drugs can regulate the immunity by interfering with immune cell regulation, pro-inflammatory cytokines’ synthesis, and gene expression. It is believed that the balance between pro-inflammatory cytokines (IL-2, IL-6, IL-8, IL-1*β*, IFN-*γ*, TNF*α*) and anti-inflammatory cytokines (IL-4, IL-10, TGF*β*) is a key factor in inflammation and immune response homeostasis, which underlies many diseases^5^. In addition, the inflammatory signaling regulation is aided by their capacity to alter the expression of number of pro-inflammatory genes, including multiple cytokines, nitric oxide synthases, lipoxygenase, and cyclooxygenase, as well as to their anti-oxidant properties like ROS (reactive oxygen species) scavenging activities^6,7^. However, immunosuppressive medications have many disadvantages and suffer from a lot of side effects as it should be administrated for long term, accompanied by adverse metabolic disturbances and toxicities, in addition to the high risk of infection, cancer incidence and lack specificity^8^.

Epidemiological evidence indicates that dietary patterns have a significant impact on inflammatory processes. Primarily consuming fruits, vegetables, and whole grains is the best way to get valuable molecules like polyphenols, resveratrol, epigallocatechin gallate, capsaicin and curcumin. These secondary plant metabolites have powerful anti-inflammatory characteristics and can modulate the signaling pathways that modify how pro-inflammatory genes like cyclooxygenase, lipoxygenase and phospholipase A2, are expressed^9,10^. In addition to observational research, there is evidence from human intervention trials that several plant diets may have anti-inflammatory properties. At the level of bioactive substances present in plant diets, carotenoids and flavonoids in particular seem to affect inflammatory as well as immunological processes. For example, a randomized, double-blind, controlled clinical trial was carried out on herbal formulation consisted of “turmeric extract, ginger, and black pepper” versus Naproxen drug for chronic knee osteoarthritis and the results demonstrated that ginger which is the most prevalent herbal product used, with active ingredients like gingerols, can reduce the inflammation and therefor relieve the pain of osteoarthritis^11^, where turmeric with curcumin as the most active component was found to be a potent anti-inflammatory agent and can reduce the complications of rheumatoid arthritis^12,13^. The results of studies also, indicate the therapeutic effects of black pepper in reducing inflammation^14^ due to the alkaloid called *pyrene*. In conclusion, there is strong evidence that plant diets and their non-nutritive by-products influence inflammatory and immune responses^15^. So that, introducing immunomodulatory reagents from natural plant sources will have great impact on drug discovery as this will provide safer bioavailable candidates with decreased production costs. Moreover, the discovered compounds will not have the undesirable side effects reported for the synthetic chemicals currently available. Even so, herbal medications are expected to be quite complex taking in consideration their chemical components, which may interact with a number of protein targets to have a therapeutic effect^16^. Such great complexity also elicits numerous pharmacodynamics interactions such as synergistic effects^17^, that arise between these various ingredients, which makes it challenging to pinpoint the precise mechanism of action of such complex substances.

Recent years have seen the successful use of network pharmacology for the building and visualization of disease-gene-target drug networks, which can aid in the assessment of the molecular mechanisms of drugs from a multi-dimensional perspective^18–20^. Given that medicinal plants are multi-component and the network pharmacology approach emphasizes the idea of “network target, multicomponent therapeutics,” in a holistic manner analogous to the complex matrices of medicinal herbals, this strategy is thought to be appropriate for understanding the mechanism of action of medicinal plants^21–24^. Network pharmacology can be used to forecast the protein targets of the active ingredients of the plants and, consequently, the disease pathways that will be affected. Predicting the primary active ingredients and probable target genes of medicinal plants involves the application of multiple targets identification process using network pharmacological analysis, which is being employed more and more^25–27^.

The aim of this work is to comprehend the mechanism of the immunomodulatory activity of some natural plant sources via network pharmacology analysis. The identification of protein targets and the related pathways followed by gene enrichment analysis were carried out. In vitro anti-inflammatory testing and docking analysis were further performed to verify the potential immunomodulatory activity of natural plant sources extract. This study displays deeper insights about natural plant sources molecular mechanisms of action in the control of autoimmune disorders utilizing an integrated strategy of network pharmacology, docking analysis and in vitro testing.

## Result and discussion

An in-house database of 2154 phytochemical constituents was constructed depending on a previous literature analysis of the chemical makeup of 32 selected immunomodulatory plants.

### ADME screening of the database of immunomodulatory plants

The ADME properties of the phytochemical constituents were evaluated through the use of the QikProp module, which determine some of the physiochemical characteristics that indicate a compound drug-likeness. The Lipinski rule of five served as a summary of these physiochemical characteristics. According to Lipinski's rule of five, a compound with reputed biological activity is deemed active (having satisfactory absorption and/or permeation) if it has a molecular weight less than 500 Da, fewer than 10 hydrogen-bond acceptors (Hacc), fewer than 5 hydrogen-bond donors (Hdon), 10 or fewer rotatable bonds (RBN), and has a measured log P (ClogP) less than five^28^. After filtration of database, only chemicals that obeyed at least three of the aforementioned characteristics were retained. Oral bioavailability (OB) of phytoconstituents was also estimated^29^. It shows how much of a pharmacological dosage taken orally still has an unaltered effect at the therapeutic site of action. In the database, only compounds with OB ≥ 30% were kept. All the database compounds that satisfied the specified requirements were maintained for further network pharmacology-based analysis (Table S1).

### Identification of target proteins of the selected immunomodulatory plants constituents using network pharmacology

Due to the extravagant cost of in-laboratory screening of numerous plants along with their complicated constituents, an *in-silico* analysis approach was tried in this study to provide a quick, effective and high throughput technique to identify the potential immunomodulatory molecular targets of the constructed phytochemicals database. A constituent-target (C-T) network dependent on 600 plant constituents and putative targets was created utilising the screening results received from the STITCH 5.0 public database in order to uncover the mechanisms of action of the chosen plant constituents and immunomodulatory-associated target genes. STITCH is a huge database that holds a big data on chemical interactions. It connects 1.5 million genes across 373 genomes with over than 68 000 compounds, including 2200 drugs, and offers information on their interactions^30^. The function of each discovered target gene and its relationship to immunomodulation were determined using the Universal Protein Resource database (UniProt). In-depth information about more than 550 000 proteins and their functions can be found in UniProt, a resource for protein sequences and functional annotation^31^. The interactions between chemicals and genes are given "combined scores" in the STITCH 5.0 database, with stronger interactions displaying higher scores. In this investigation, only substances with interaction scores greater than 0.4 were kept (Table 1).

**Table 1:** Potential protein targets of the selected plants compounds.

UniProtKB entry ID	Gene ID	Protein name	Interacting compound(s)	combined interacting score
P33527	ABCC1	Multidrug resistance-associated protein 1	Nobiletin	0.8
			Bisdemethoxycurcumin	0.8
			5,7,3′,4′-tetramethoxyflavone	0.7
			Chrysoeriol	0.783
P31749	AKT1	RAC-alpha serine/threonine-protein kinase	Urolithin A	0.8
			Diallyl trisulfide	0.84
			Apigenin	0.876
P42574	CASP3	Caspase-3	Thymoquinone	0.822
			Diallyl trisulfide	0.945
			Guaiazulene	0.7
			Oleanolic acid	0.735
			Apigenin	0.947
P24385	CCND1	G1/S-specific cyclin-D1	Silibinin	0.824
P24941	CDK2	Cyclin-dependent kinase 2	Luteoin	0.942
			Geraniol	0.8
P35222	CTNNB1	Catenin beta-1 (Beta-catenin)	Silibinin	0.831
P01133	EGF	Epidermal growth factor	Prunetin	0.8
P01100	FOS	Proto-oncogene c-Fos	Luteolin	0.944
P28223	HTR2A	5-hydroxytryptamine receptor 2A	Glabrene	0.8
			2,4-dihydroxybenzoic acid	0.8
			Homovanillic acid	0.862
P05362	ICAM1	Intercellular adhesion molecule 1	Glabridin	0.8
P22301	IL10	Interleukin-10	Aspirin	0.954
P05112	IL4	Interleukin-4	Ellagic acid	0.834
P05412	JUN	Transcription factor AP-1	Luteolin	0.946
P28482	MAPK1	Mitogen-activated protein kinase 1	Allicin	0.7
Q16539	MAPK14	Mitogen-activated protein kinase 14	Ar-turmerone	0.8
P27361	MAPK3	Mitogen-activated protein kinase 3	Allicin	0.7
P45983	MAPK8	Mitogen-activated protein kinase 8	Luteolin	0.951
P08253	MMP2	72 kDa type IV collagenase	Nobiletin	0.8
			Diallyl trisulfide	0.943
			Gallic acid	0.951
			Kaempferol	0.815
			Galangin	0.8
			Silibinin	0.825
P14780	MMP9	Matrix metalloproteinase-9	Nobiletin	0.818
			Vanillin	0.8
			Luteolin	0.949
			Apigenin	0.842
			Aspirin	0.961
			Silibinin	0.855
P42345	MTOR	Serine/threonine-protein kinase mTOR	Galangin	0.734
P01106	MYC	Myc proto-oncogene protein	Nobiletin	0.8
P29475	NOS1	Nitric oxide synthase, brain	Bisdemethoxycurcumin	0.596
			Thymoquinone	0.596
			Glabridin	0.786
			Oleocanthal	0.786
			Caffeic acid phenethyl ester	0.406
P35228	NOS2	Nitric oxide synthase, inducible	Bisdemethoxycurcumin	0.733
			Thymoquinone	0.733
			Allicin	0.733
			Oleocanthal	0.613
			Caffeic acid phenethyl ester	0.425
P29474	NOS3	Nitric oxide synthase, endothelial	Bisdemethoxycurcumin	0.596
			Diallyl trisulfide	0.938
			Aspirin	0.957
			Oleocanthal	0.613
			Caffeic acid phenethyl ester	0.406
			Ellagic acid	0.828
			Sesamol	0.817
P60484	PTEN	Phosphatidylinositol 3,4,5-trisphosphate 3-phosphatase and dual-specificity protein phosphatase PTEN	Thymoquinone	0.85
P35354	PTGS2	Prostaglandin G/H synthase 2	Catechin	0.833
			Thymoquinone	0.8
			Diallyl trisulfide	0.824
			Aspirin	0.999
			Sesamol	0.82
			Maslinic acid	0.8
			Apigenin	0.877
P04049	RAF1	RAF proto-oncogene serine/threonine-protein kinase	Silibinin	0.822
P12931	SRC	Proto-oncogene tyrosine-protein kinase Src	Carnosic acid	0.8
P04637	TP53	Cellular tumor antigen p53	Aspirin	0.97
			Chrysin	0.722
			Silibinin	0.85
			Apigenin	0.868
O75762	TRPA1	Transient receptor potential cation channel subfamily A member 1	1,4-cineole	0.829
			Eugenol	0.873
			Allicin	0.962
			Zingerone	0.43
			1,8-cineole	0.837
P19320	VCAM1	Vascular cell adhesion protein 1	Galangin	0.8
P15692	VEGFA	Vascular endothelial growth factor A	Nobiletin	0.8
			Carnosic acid	0.8

**Figure 1:**
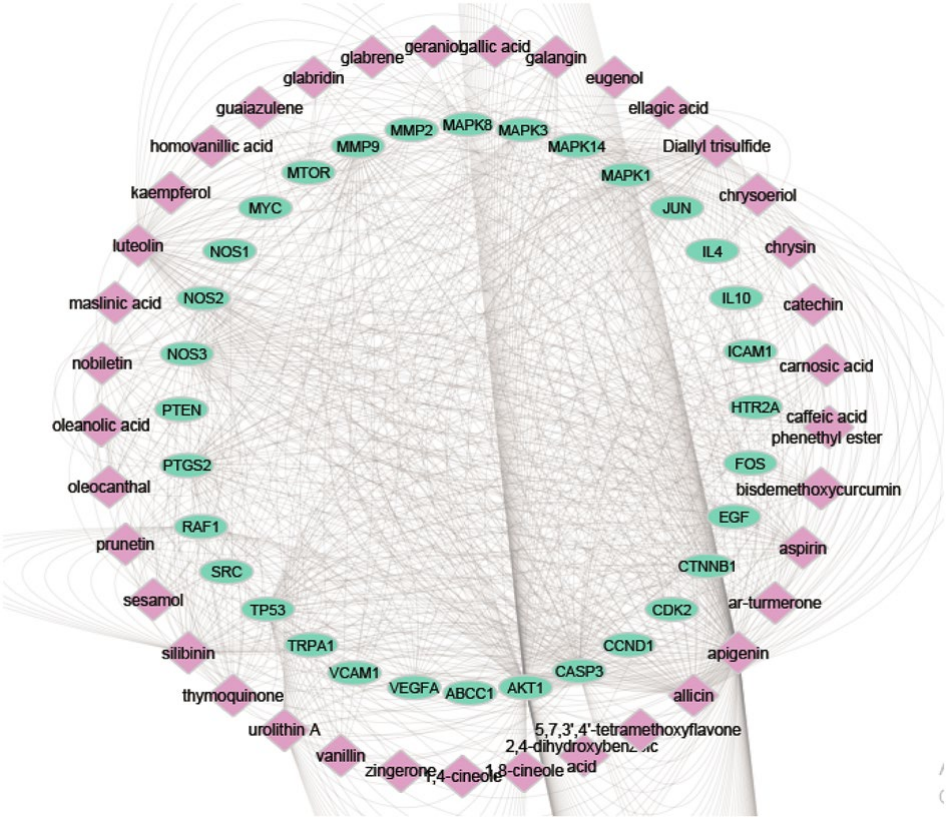
Network of compound-target gene (C-T) interactions for the selected plants constituents by linking 37 constituents and 32 targets.

**Figure 2:**
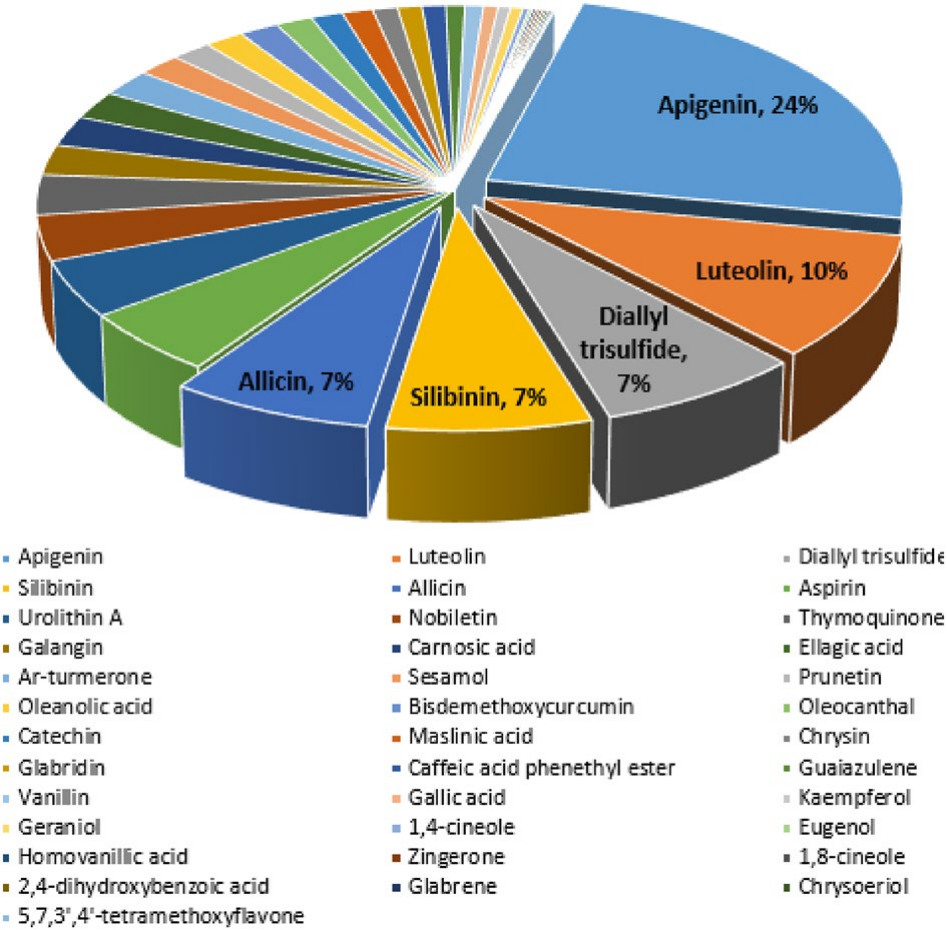
The distributions % of the 759 C-T interactions on the selected plant constituents.

A total of 69 nodes (37 constituents and 32 targets) and 759 edges made up the constructed constituent-target (C-T) network (Fig. 1), with an average of 2.435 targets for each constituent illustrating the multi-target property of the investigated compounds. The C-T network revealed that a single constituent can interact with various targets, and that targets are frequently linked to a number of different components at once. Network topological analysis was used to examine the distribution of C-T interactions on the 37 ingredients, and the results showed that apigenin had the highest percentage of C-T interactions (24%) followed by luteolin (10%), diallyl trisulfide (7.34%), silibinin (7.2%), and allicin (7.1%), respectively (Fig. 2, Table S2).

**Figure 3:**
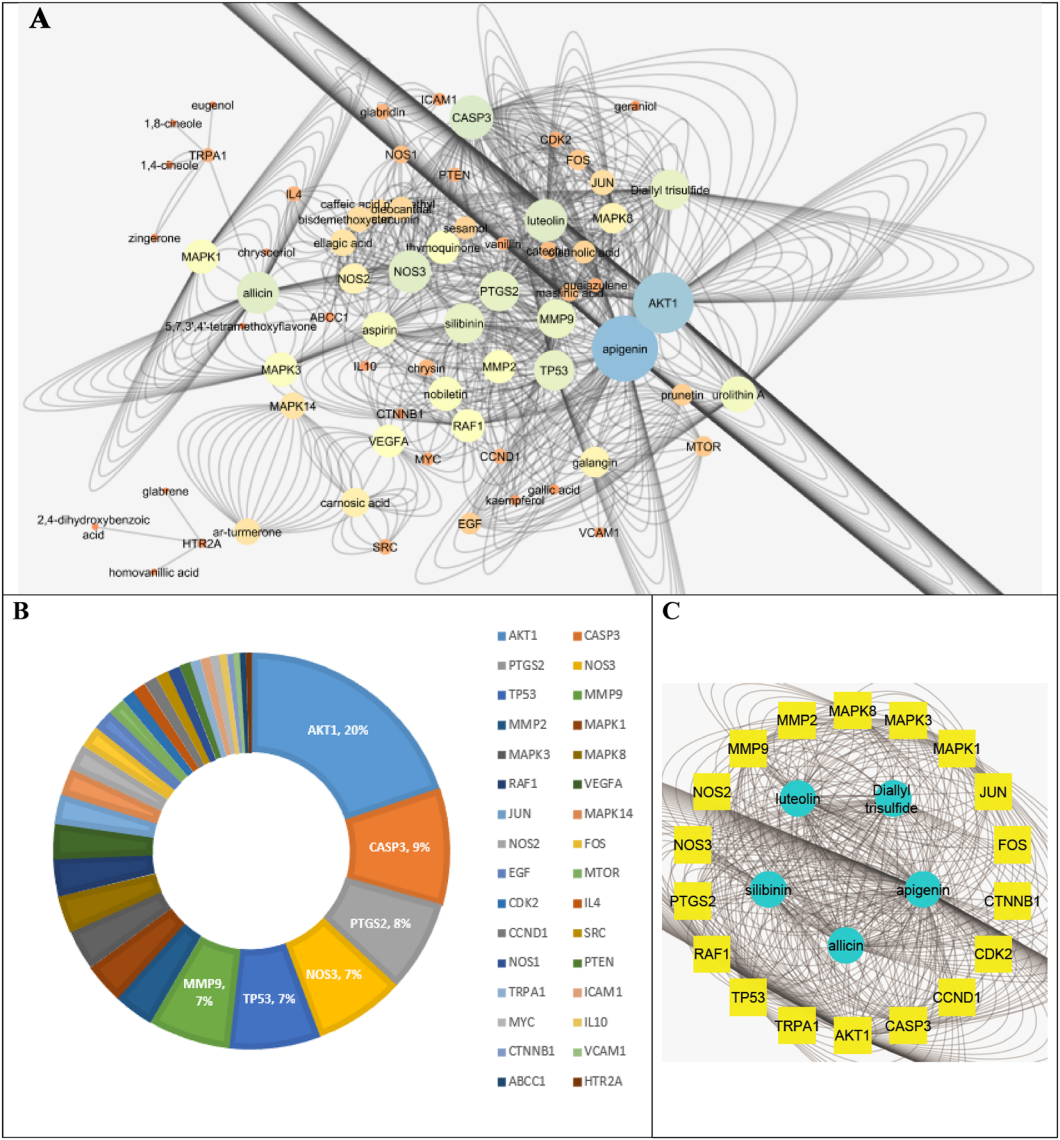
C-T network expressed as nodes of different sizes according to their interactions scores in the network (**A**), the distributions % of the C–T interactions on the identified immunomodulatory proteins (**B**) and top scoring C-T genes network (**C**).

**Figure 4:**
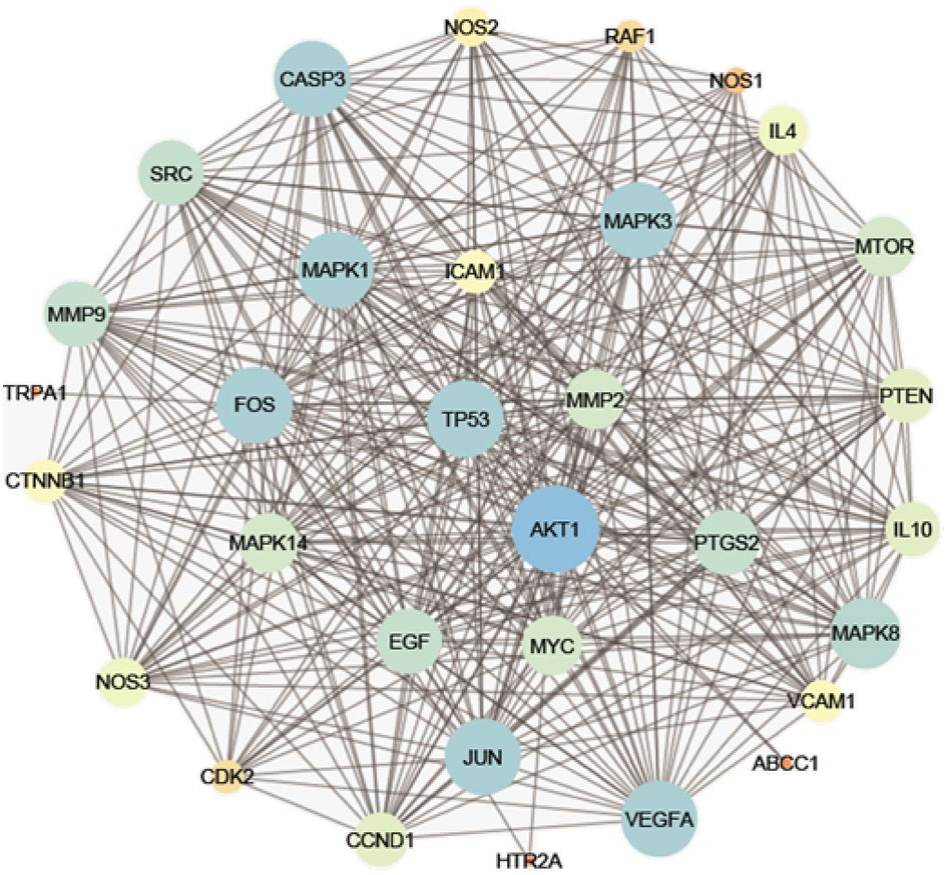
Protein–protein interaction (PPI) network of identified targets.

Table 1 displays the discovered target genes in the network. The targeted genes AKT1 (20%), CASP3 (9%), PTGS2 (9%), NOS3 (7%), TP53 (7%) and MMP9 (7%) were found to be the most enriched ones by possessing the highest combined scores and therefore the highest percentage of interactions with the network constituents, suggesting that they may be the key nodes in that network (Fig. 3A,B). Investigation of the C-T subnetwork among the 5 top scoring constituents displayed the target genes AKT1, TP53, CASP3, NOS3 and MMP9 to be the most enriched genes (Fig. 3C). It is widely recognised that AKT1 (protein kinase B *α* or RAC-alpha serine/threonine-protein kinase) is a crucial signaling node in controlling the activation of adaptive immune cells^32^ because it controls a variety of activities such as metabolism, cell survival, growth, proliferation, and angiogenesis^33^. Additionally, it has been shown to have substantial anti-inflammatory effects through inhibiting NF-*κ*B-mediated transcription^34^. Meanwhile, CASP3 (Caspase-3) is implicated in the activation cascade of caspases necessary for apoptosis execution^35^. Prostaglandin-endoperoxide synthase 2 (PTGS2) is an essential enzyme for inflammatory prostaglandins biosynthesis and production^36^, while tumor suppressor protein (TP53) affects the innate immune system by secreting factors that modify macrophage activity to prevent tumour development. Additionally, it inhibits some pro-inflammatory proteins like NF-*κ*B and STAT3, to support tissue homeostasis and prevent overreacting immune systems. Moreover, nitric oxide synthase-3 (NOS-3) is hypothesised to play anti-apoptotic roles and engaged in the suppression of genes implicated in cell proliferation. It also reduces the expression of several cytokines such as INF-*γ*, IL-1*β*, IL-6 and TNF-*α*, in a variety of immune cells including monocytes, eosinophils and lymphocytes. The nitrosylation of several transcription factors, including JAK/STAT and NF-*κ*B, mediates this action^37^. On the other hand, TP53 helps to recognise non-self-antigens, which in turn triggers anti-tumor immunity through a variety of pathways^38–40^. Matrix metalloproteinase 9 (Mmp9) is a secreted gelatinase that participates in the remodelling of the extracellular matrix by degrading the components of the extracellular matrix. It also controls the pro-inflammatory cytokines TNF-*γ* and IL-1 to control the inflammatory response^41^. A protein–protein network revealed significant gene–gene interactions (Fig. 4) indicating a high extent of synergism between them.

**Figure 5:**
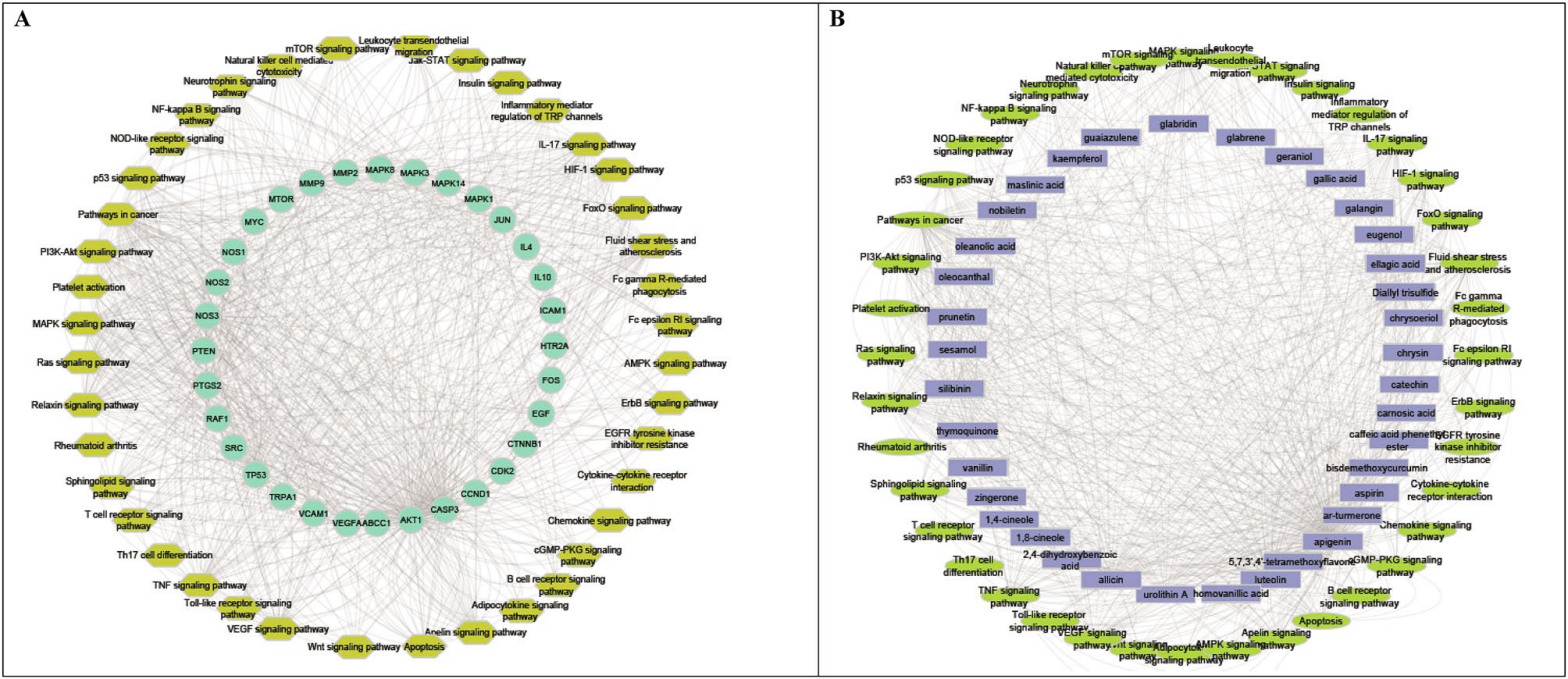
Gene-pathway network (genes are presented in green color, pathways are presented in yellow color) (**A**), compound-pathway network (compounds are presented in purple color, pathways presented in light green color) (**B**).

KEGG (Kyoto Encyclopedia of Genes and Genomes) is a database tool for deriving high-level biological system functions and applications from molecular-level data, particularly from wide-ranging molecular datasets produced by genome sequencing and other high-throughput experimental techniques^42–44^. To understand the signaling pathways and roles of the identified target genes, KEGG pathways functional enrichment analysis was carried out (Table 2). It can be seen from the KEGG analysis (Table 2 and Fig. 5A) that the target genes interact with 40 immunomodulatory-associated pathways, with pathways in cancer having the largest number of observed genes and the smallest false discovery rate, followed by fluid shear stress and atherosclerosis, relaxin signaling pathway, IL-17 signaling pathway and FoxO signaling pathway. To further clarify the receptor-ligand interactions associated to the disease, the constituent-pathway network (Fig. 5B) was built to connect the constituents with the signaling pathway.

**Table 2:** KEGG pathway analysis of potential target genes functions^42–44^.

#Term ID	Term description	Observed gene count	False discovery rate	Matching proteins in network (labels)
hsa05200	Pathways in cancer	31	8.49E-16	MAPK1, HMOX1, MMP2, CCND1, IL4, RAF1, MAPK3, EGF, CDK2, TP53, PPARG, PTGER1, FOS, CYCS, CASP3, NQO1, NOS2, CTNNB1, MTOR, PTGS2, JUN, PTEN, MMP9, PIM1, MAPK8, NFE2L2, GSTP1, ESR1, AKT1, VEGFA, MYC
hsa05418	Fluid shear stress and atherosclerosis	18	1.21E-14	HMOX1, MMP2, MAPK14, ICAM1, TP53, VCAM1, NOS3, FOS, NQO1, CTNNB1, JUN, MMP9, SRC, MAPK8, NFE2L2, GSTP1, AKT1, VEGFA
hsa04926	Relaxin signaling pathway	15	1.08E-11	MAPK1, MMP2, MAPK14, RAF1, MAPK3, NOS3, FOS, NOS2, JUN, MMP9, SRC, MAPK8, AKT1, NOS1, VEGFA
hsa04657	IL-17 signaling pathway	12	5.05E-10	MAPK1, MAPK14, IL4, MAPK3, CCL11, FOS, CASP3, PTGS2, S100A8, JUN, MMP9, MAPK8
hsa04068	FoxO signaling pathway	13	1.33E-09	MAPK1, CCND1, MAPK14, CAT, RAF1, MAPK3, EGF, CDK2, PLK1, PTEN, MAPK8, IL10, AKT1
hsa04210	Apoptosis	13	1.76E-09	MAPK1, RAF1, MAPK3, BCL2A1, TP53, FOS, CYCS, CASP3, PARP1, MCL1, JUN, MAPK8, AKT1
hsa04668	TNF signaling pathway	12	1.93E-09	MAPK1, MAPK14, MAPK3, ICAM1, VCAM1, FOS, CASP3, PTGS2, JUN, MMP9, MAPK8, AKT1
hsa04012	ErbB signaling pathway	10	2.50E-08	MAPK1, RAF1, MAPK3, EGF, MTOR, JUN, SRC, MAPK8, AKT1, MYC
hsa04370	VEGF signaling pathway	9	2.50E-08	MAPK1, MAPK14, RAF1, MAPK3, NOS3, PTGS2, SRC, AKT1, VEGFA
hsa04115	p53 signaling pathway	9	6.38E-08	CCND1, CDK2, TP53, CYCS, CASP3, PTEN, IGFBP3, CDK1, CHEK1
hsa04660	T cell receptor signaling pathway	10	9.09E-08	MAPK1, MAPK14, IL4, RAF1, MAPK3, FOS, BCL10, JUN, IL10, AKT1
hsa01521	EGFR tyrosine kinase inhibitor resistance	9	1.51E-07	MAPK1, RAF1, MAPK3, EGF, MTOR, PTEN, SRC, AKT1, VEGFA
hsa04071	Sphingolipid signaling pathway	10	3.04E-07	MAPK1, MAPK14, RAF1, MAPK3, TP53, NOS3, PTEN, MAPK8, ABCC1, AKT1
hsa04010	MAPK signaling pathway	14	6.84E-07	MAPK1, MAPK14, RAF1, MAPK3, EGF, TP53, FOS, CASP3, JUN, RPS6KA3, MAPK8, AKT1, VEGFA, MYC
hsa04066	HIF-1 signaling pathway	9	7.67E-07	MAPK1, HMOX1, MAPK3, EGF, NOS3, NOS2, MTOR, AKT1, VEGFA
hsa04151	PI3K-Akt signaling pathway	15	8.03E-07	MAPK1, CCND1, IL4, RAF1, MAPK3, EGF, CDK2, TP53, NOS3, MTOR, MCL1, PTEN, AKT1, VEGFA, MYC
hsa04371	Apelin signaling pathway	10	8.30E-07	MAPK1, CCND1, RAF1, UCP1, MAPK3, NOS3, NOS2, MTOR, AKT1, NOS1
hsa04722	Neurotrophin signaling pathway	9	2.48E-06	MAPK1, MAPK14, RAF1, MAPK3, TP53, JUN, RPS6KA3, MAPK8, AKT1
hsa04630	Jak-STAT signaling pathway	10	3.50E-06	CCND1, IL4, RAF1, EGF, MTOR, MCL1, PIM1, IL10, AKT1, MYC
hsa04611	Platelet activation	9	3.58E-06	MAPK1, MAPK14, MAPK3, PTGIR, NOS3, PTGS1, SRC, TBXA2R, AKT1
hsa04664	Fc epsilon RI signaling pathway	7	6.10E-06	MAPK1, MAPK14, IL4, RAF1, MAPK3, MAPK8, AKT1
hsa04659	Th17 cell differentiation	8	8.12E-06	MAPK1, MAPK14, IL4, MAPK3, FOS, MTOR, JUN, MAPK8
hsa04662	B cell receptor signaling pathway	7	8.38E-06	MAPK1, RAF1, MAPK3, FOS, BCL10, JUN, AKT1
hsa04064	NF-kappa B signaling pathway	7	3.68E-05	CSNK2A1, ICAM1, BCL2A1, VCAM1, PARP1, PTGS2, BCL10
hsa04620	Toll-like receptor signaling pathway	7	6.25E-05	MAPK1, MAPK14, MAPK3, FOS, JUN, MAPK8, AKT1
hsa04022	cGMP-PKG signaling pathway	8	0.00013	MAPK1, RAF1, MAPK3, SLC25A4, NOS3, SLC25A5, SLC25A6, AKT1
hsa04062	Chemokine signaling pathway	8	0.00028	MAPK1, RAF1, MAPK3, PPBP, CCL11, SRC, HCK, AKT1
hsa04750	Inflammatory mediator regulation of TRP channels	6	0.00028	MAPK14, TRPA1, SRC, MAPK8, HTR2A, VR1
hsa04310	Wnt signaling pathway	7	0.0004	CSNK2A1, CCND1, TP53, CTNNB1, JUN, MAPK8, MYC
hsa04150	mTOR signaling pathway	7	0.00048	MAPK1, RAF1, MAPK3, MTOR, PTEN, RPS6KA3, AKT1
hsa04920	Adipocytokine signaling pathway	5	0.0006	MTOR, MAPK8, PPARA, ADIPOQ, AKT1
hsa04670	Leukocyte transendothelial migration	6	0.0007	MMP2, MAPK14, ICAM1, VCAM1, CTNNB1, MMP9
hsa04910	Insulin signaling pathway	6	0.0016	MAPK1, RAF1, MAPK3, MTOR, MAPK8, AKT1
hsa04666	Fc gamma R-mediated phagocytosis	5	0.0017	MAPK1, RAF1, MAPK3, HCK, AKT1
hsa04621	NOD-like receptor signaling pathway	6	0.004	MAPK1, NAMPT, MAPK14, MAPK3, JUN, MAPK8
hsa04014	Ras signaling pathway	7	0.0043	MAPK1, RAF1, MAPK3, EGF, MAPK8, AKT1,VEGFA
hsa04152	AMPK signaling pathway	5	0.005	CCND1, PPARG, MTOR, ADIPOQ, AKT1
hsa04650	Natural killer cell mediated cytotoxicity	5	0.0057	MAPK1, RAF1, MAPK3, ICAM1, CASP3
hsa05323	Rheumatoid arthritis	4	0.008	ICAM1, FOS, JUN, VEGFA
hsa04060	Cytokine-cytokine receptor interaction	6	0.0283	IL4, EGF, PPBP, CCL11, IL10, VEGFA

The PubMed database was searched for publications linking the hit compounds to the various immunomodulatory pathways in order to validate the findings from the network pharmacology-based study (Table 3). For instance, apigenin (NF-*κ*B and COX-2 inhibitor), was found to be valuable in lupus therapy and also for preventing inflammation in other Th17-mediated inflammatory diseases like Crohn’s disease, rheumatoid arthritis, and psoriasis as well as in prevention of inflammation-based tumors overexpressing COX-2 in colon and breast as it was found to inhibit the autoantigen-presenting and stimulatory activities of the APCs (antigen presenting cells), which are required for the activation and development of lupus-associated Th1 and Th17 cells as well as B cells and causes the overactive lupus APCs, B cells, and T cells to undergo apoptosis, most likely by suppressing the expression of NF-*κ*B-regulated anti-apoptotic molecules, particularly COX-2 and c-FLIP, which are chronically overexpressed by the lupus immune cells^45–47^. In addition, luteolin was reported to inhibit the overexpression of inflammatory mediators and catabolic factors, probably via preventing NF-*κ*B activation. Additionally, it prevents T cell growth and their AKT/mTOR signaling. Thus, luteolin is an emerging immunosuppressant as a novel mTOR inhibitor. It also ameliorates inflammation and Th1/Th2 imbalance by regulating the TLR4/NF-*κ*B pathway^48–50^. Meanwhile, diallyl trisulfide (DAT) were proved to have a potential anti-inflammatory activity by possessing an antagonistic action on TLR4 (Toll-like receptor 4/nuclear factor-*κ*B pathway) as it inhibits the production of myeloid differentiation factor 88. DAT was reported also to increase the stabilization of I*κ*B*α* by preventing its phosphorylation by I*κ*B kinase (IKK) complex, and inhibit the NF-*κ*B transcriptional activites^51,52^. Moreover, silibinin was declared to dramatically decrease the demyelination and inflammation signs in experimental autoimmune encephalomyelitis by upregulating the release of the anti-inflammatory Th2 cytokines and downregulating the pro-inflammatory Th1 cytokines. It also showed anti-carcinogenic and anti-inflammatory properties as a result of NF-*κ*B transcription factor suppression. Additionally, through ER*β* binding, silibinin triggers apoptosis, slows proliferation, and inhibits the production of the pro-inflammatory cytokines TNF-*α* and IL-17 in CD4 + T cells^53,54^. Also, allicin was proclaimed to suppress the chemokines IL-8, IP-10, and MIG, as well as the release of IL-1*β* from intestinal epithelial cells. This action was at least partially mediated by down-regulating the mRNA levels and the involved inhibition of activation of the NF-*κ*B pathway^55^.

**Table 3:** Literature survey summary on the top scoring immunomodulatory plant constituents used as anti-inflammatory agents.

Compound	Model	Mechanism	Reference
Apigenin	Induced systemic lupus erythematosus in the (SWR × NZB) F1 (SNF1) mouse model	Inhibits autoantigen-presenting and stimulatory activities of the APCs (antigen presenting cells), which are required for the activation and development of lupus-associated Th1 and Th17 cells as well as B cells Causes the overactive lupus APCs, B cells, and T cells to undergo apoptosis, most likely by suppressing the expression of NF-*κ*B-regulated anti-apoptotic molecules, particularly COX-2 and c-FLIP, which are chronically overexpressed by the lupus immune cells Increasing the bioavailability of apigenin (NF-*κ*B and COX-2 inhibitor), may be valuable in lupus therapy and also for preventing inflammation in other Th17-mediated inflammatory diseases like Crohn disease, rheumatoid arthritis, and psoriasis as well as in prevention of inflammation-based tumors overexpressing COX-2 (colon, breast)	PMID: 19,405,952
	LPS-Induced NF-*κ*B Activity in mice lungs	Effectively modulates NF-*κ*B activity in the lungs, suggesting its ability to exert specific immune-regulatory effects	PMID: 26,938,530
	Human and murine autoreactive T cells	Downregulates P2X7/NFKB pathway, inhibits IL1b, MMP (3,1,13) and ADAMTS-5 Reduces T cells proliferation, producing IFN-*ɣ*, ROS and impedes phagocytosis	PMID: 30,229,507
Luteolin	Human and murine autoreactive T cells	Strongly inhibits human and murine T cells and FN-*ɣ* secretion	PMID: 15,276,069
	Interleukin (IL)-1*β* stimulated rat chondrocytes and a monosodium iodoacetate (MIA)-induced model of osteoarthritis	Inhibits the overexpression of inflammatory mediators and catabolic factors, probably via preventing NF-*κ*B activation Can reduce cartilage degradation in osteoarthritic rats caused by MIA	PMID: 30,551,412
	MG-induced apoptosis in PC12 cells	Pre-treatment with luteolin can dramatically increase cell viability, inhibit the activation of the mTOR/4E-BP1 signaling pathway, reduce methylglyoxal (MG)-induced apoptosis, and decrease pro-apoptotic proteins such as Cytochrome C, Bax and caspase-3	PMID: 28,801,605
	C57BL/6 and BALB/c mice	Reduces the frequency of effector T cells, induces CD4 + Foxp3 + Tregs, and inhibits DC maturation while suppressing allograft rejection Additionally, it prevents T cell growth and their AKT/mTOR signaling. Thus, luteolin is an emerging immunosuppressant as a novel mTOR inhibitor	PMID: 31,732,527
	Allergic rhinitis rat model	Ameliorates inflammation and Th1/Th2 imbalance by regulating the TLR4/NF-*κ*B pathway	PMID: 33,900,898
Diallyl trisulfide	KSHV-positive PEL cell lines (BC2, BC3, BCBL1 and HBL6)	DAT increases the stabilization of IκBα by preventing its phosphorylation by I*κ*B kinase (IKK) complex,and inhibits the NF-*κ*B transcriptional activites in PEL cells Additionally, DAT causes TRAF6 to be degraded by proteasomes and, through downregulating TRAF6, DAT prevents IKK*β*-phosphorylation	PMID: 26,647,777
	Mmurine RAW 264.7 macrophage cell line	DATS has an antagonistic action on TLR4 as it inhibits the production of myeloid differentiation factor 88 and the LPS binding to macrophages Additionally, in LPS-induced RAW 264.7 macrophages, inhibiting TLR4 signaling with the particular TLR4 signaling inhibitors, CLI-095, improved the anti-inflammatory potential of DATS	PMID: 25,500,681
Silibinin	Healthy subjects as well as patients with active RA	Through ERβ binding, silibinin triggers apoptosis, slows proliferation, and inhibits the production of the pro-inflammatory cytokines TNF-*α* and IL-17 in CD4 + T cells from both female and male healthy participants Acts as an epigenetic modifier that suppresses the expression of miR-155, which is important for the pathophysiology of RA	PMID: 30,174,672
	MS animal model	Silibinin dramatically decreases the demyelination and inflammation signs in experimental autoimmune encephalomyelitis by upregulating the release of the anti-inflammatory Th2 cytokines and downregulating the pro-inflammatory Th1 cytokines Shows anti-carcinogenic and anti-inflammatory properties as a result of NF-*κ*B transcription factor suppression	PMID: 18,038,905
Allicin	HT-29 (ATCC HTB38) and Caco-2 (ATCC HTB27) cells	The chemokines IL-8, IP-10, and MIG, as well as the release of IL-1*β* from intestinal epithelial cells, are all suppressed by allicin. This action was at least partially mediated by down-regulating the mRNA levels and the involved inhibition of activation of the NF-*κ*B pathway	PMID: 15,380,914

### Target proteins of the selected plants using combined network pharmacology

**Figure 6:**
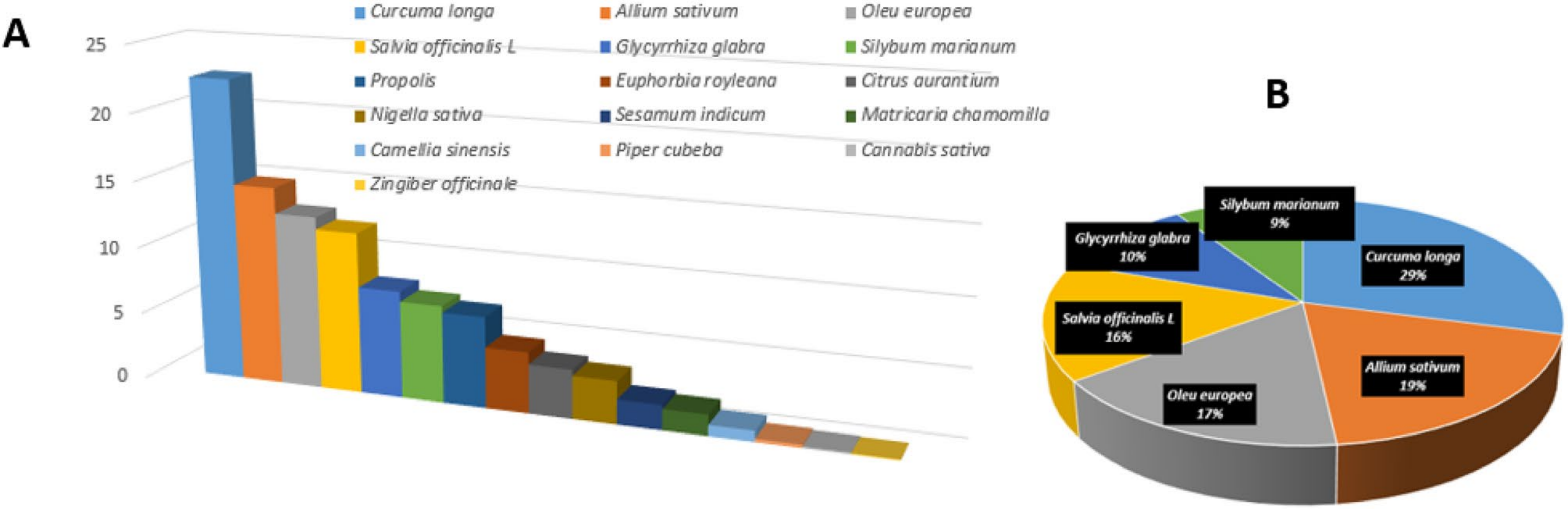
Bar chart representing the percentage of distributions of the 759 C-T interactions on the selected plants in the database (**A**) and pie chart illustrating the plants with the highest percentage (**B**).

After looking at the 759 C-T interaction distributions on the selected plants, a combined plant-constituent-target-pathway network was created. The cytoscape combined score of C-T interactions was used to rate the plants. Figure 6 illustrates that *Curcuma longa* (29%), *Allium sativum* (19%), *Oleu europea* (17%), *Salvia officinalis L* (16%), *Glycyrrhiza glabra* (10%) and *Silybum marianum* (9%) had the highest number of P-C-T-P interactions which would suggest that these plants have more active substances that can act as immunomodulatory agents. In reality, there are numerous reports on the anti-inflammatory action of *Curcuma longa* in traditional medicine. In Indian medicinal system, *Curcuma longa* is used in wound healing and as an anti-inflammatory and anticancer plant^56^. Meanwhile, *Allium sativum* has been utilized traditionally since ancient times due to its biological properties including anti-tumour, anti-inflammatory, antioxidant, tuberculosis, arthritis, rhinitis, bronchitis, and immunomodulatory activities^57,58^. Moreover, essential oil extract of *Oleu europea* fruit is applied topically in Italy to alleviate rheumatism and to boost the circulation^59^. Infusion of fresh leaves of *O. europaea* is utilised in Tunisian traditional medicine as an antioxidant, anticancer and as a remedy for various types of inflammation^60^. In addition, *Salvia officinalis L* has been used to cure rheumatism and inflammation in Latin American and Asian folk medicine^61^. It also, has been utilized to treat throat and skin inflammations in European traditional medicine^62^. Numerous investigations have been carried out in recent years to identify novel biological effects of *Salvia officinalis L* and to document its traditional usage. Numerous pharmacological actions, such as antioxidant, anticancer, anti-nociceptive, anti-inflammatory, and antimutagenic properties, have been identified by these research^63^. While *Glycyrrhiza glabra* has long been utilised traditionally in Japan as an antioxidant, anti-inflammatory, cancer preventative, and anti-arthritic agent^64,65^. Finally, *Silybum marianum* also was reported to exhibit immunomodulating and antioxidant activities by scavenging free radicals, in addition to its ability to reduce inflammation and inflammation-related pain^66^.

### Gene ontology (GO) enrichment analysis for targets

**Figure 7:**
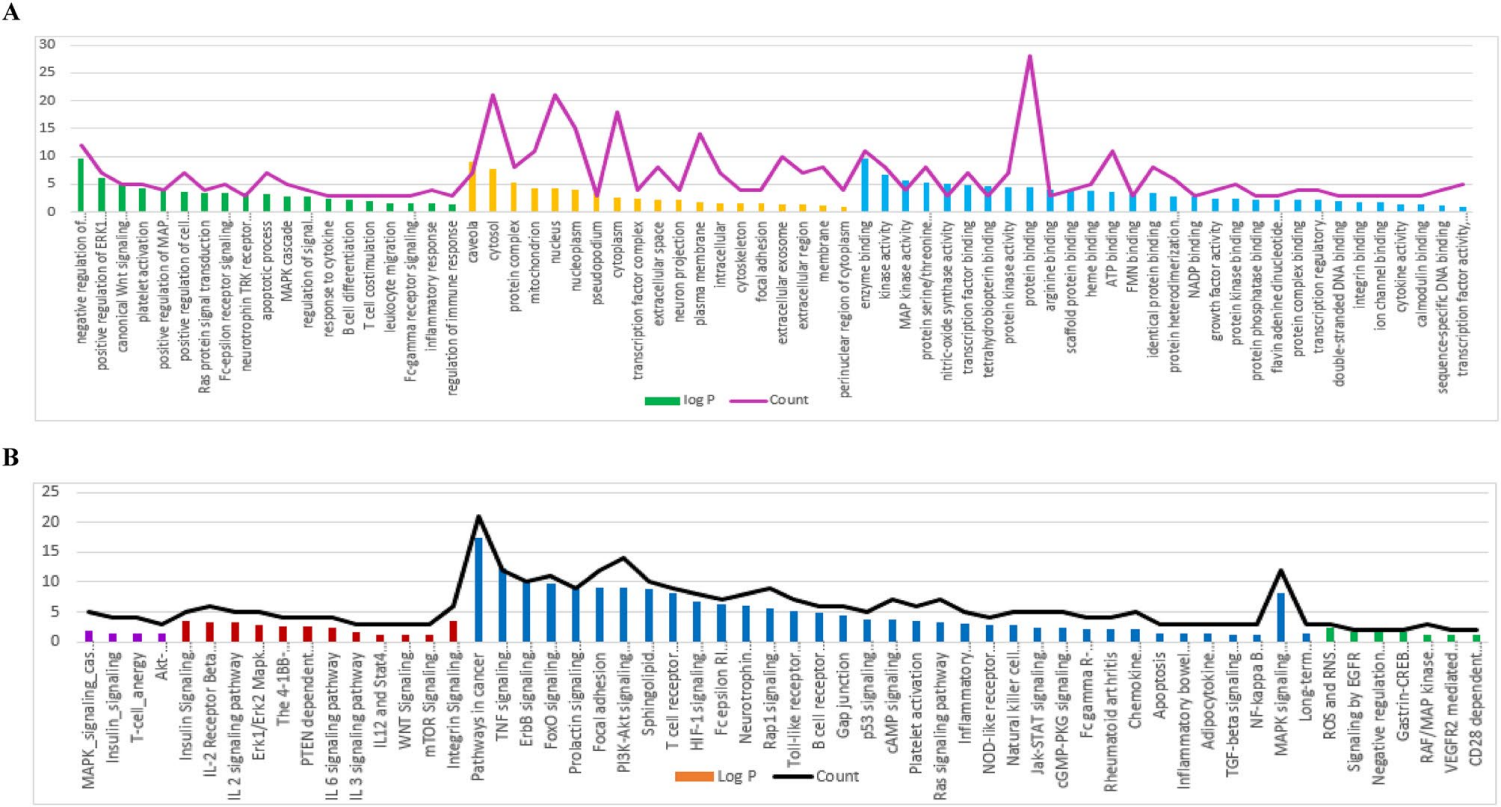
(**A**) GO enrichment analysis of discovered targets. Biological processes are colored green, cellular components are orange and molecular functions are blue. (**B**) BBID (purple), BIOCARTA (brown), KEGG (blue) and REACTOME (green) pathways analysis involved in immunomodulatory action. The order of importance was ranked by − log 10 (*P*-value) with bar chart. The number of targets stick into each term with line chart.

Gene ontology (GO) assigns several GO concepts to a single query sequence and classifies gene products into three distinct categories: biological process, cellular component, and molecular function^67^. By importing the selected targets into the DAVID bioinformatics tools and restricting the annotations to *Homo sapiens*, GO enrichment analysis was performed on the targets, revealing the most enriched pathways and GO keywords with the greatest log P value and gene counts. As demonstrated in Fig. 7A, the identified targets are linked with numerous biological processes, the most enriched ones are negative regulation of apoptotic process, positive regulation of ERK1 and ERK2 cascade, canonical Wnt signaling pathway, platelet activation and positive regulation of MAP kinase activity. The most important molecular cellular components were caveola, cytosol, protein complex, mitochondrion and nucleus. Additionally, it was shown that the most enriched molecular activities were enzyme binding, kinase activity, MAP kinase activity, protein serine/threonine kinase activity and nitric-oxide synthase activity. However, functional annotations with DAVID bioinformatics tools identified 4 BBID pathway named: MAPK_signaling_cascades, insulin-signaling, T-cell_anergy and Akt-PKB_effector_of_P13K_*in_vivo*,in addition to 12 BIOCARTA pathways named: insulin signaling pathway, IL-2 receptor beta chain in T cell activation, IL 2 signaling pathway, Erk1/Erk2 Mapk signaling pathway, The 4-1BB-dependent immune response, PTEN dependent cell cycle arrest and apoptosis, IL 6 signaling pathway, IL 3 signaling pathway, IL12 and Stat4 dependent signaling pathway in Th1 development, WNT signaling pathway, mTOR signaling pathway and integrin signaling pathway. Furthermore, 35 KEGG pathways including pathways in cancer, TNF signaling pathway, ErbB signaling pathway, FoxO signaling pathway, prolactin signaling pathway, focal adhesion and PI3K-Akt signaling pathway. In addition, 7 REACTOME pathways named ROS and RNS production in phagocytes, signaling by EGFR, negative regulation of the PI3K/AKT network, gastrin-CREB signaling pathway via PKC and MAPK, RAF/MAP kinase cascade, VEGFR2 mediated cell proliferation and CD28 dependent PI3K/Akt signaling were identified (Fig. 7B). All of these identified pathways had *P*-values less than or equal to 0.01, indicating a strong correlation with inflammation.

### Evaluation of network pharmacology analysis

The evaluation process including the three aspects—reliability, standardization, and rationality, was carried out according to the method described previously by Li^68^. The results were found to fulfil all the required criteria settled for network pharmacology analysis evaluation.

### In vitro assays of top two target proteins; Akt1 and Caspase-3

Based on network pharmacology analysis results, additional laboratory-based in vitro screening of both Akt1 and caspase-3 inhibitory activities of the four-top scoring plants; *Curcuma longa*, *Allium sativum*, *Oleu europea* and *Salvia officinalis L*, were performed utilizing colorimetric tests. The definitive goal of these in vitro investigations is to verify the network pharmacology based analysis results through assessment of some plants-targets binding affinities. Saturosporine was employed as a positive control for Akt1 inhibitory assay since it has been showed to be a strong inhibitor of Akt1 gene^69^, where Ac-DEVD-CHO, obtained from caspase 3 colorimetric assay kit supplied by Sigma, was used as a positive control for caspase-3 gene. The results demonstrated that among the screened agents, *Curcuma longa* was found to have the higher inhibitory activities on both Akt1 and caspase-3 assays with *IC*_*50*_ of 475 µg/ml ± 0.031 and 500 µg/ml ± 0.053, respectively. The Akt1 inhibitory activity of other tested inhibitors was in the order of *Allium sativum* (*IC*_*50*_ = 690 µg/ml ± 0.044) followed by *Oleu europea* (*IC*_*50*_ = 715 µg/ml ± 0.014) and finally *Salvia officinalis L* (*IC*_*50*_ = 740 µg/ml ± 0.063), respectively. Whereas, the caspase-3 inhibitory activity of other tested inhibitors was in the order of *Salvia officinalis L* (*IC*_*50*_ = 590 µg/ml ± 0.045) followed by *Allium sativum* (*IC*_*50*_ = 625 µg/ml ± 0.072) and finally *Oleu europea* (*IC*_*50*_ = 800 µg/ml ± 0.021), respectively, (Table 4).

**Table 4:** In vitro Akt1 and Caspase-3 inhibitory activity of the tested agents, where data are represented as mean of two tests ± SD.

The tested agent	Akt1	Caspase-3
	*IC*_*50*_ (µg/ml)	*IC*_*50*_ (µg/ml)
*Curcuma longa*	475 ± 0.031	500 ± 0.053
*Allium sativum*	690 ± 0.044	625 ± 0.072
*Oleu europea*	715 ± 0.014	800 ± 0.021
*Salvia officinalis L*	740 ± 0.063	590 ± 0.045
Saturosporine	0.6 (µM) ± 0.022	
Caspase-3 Inhibitor I (N-Ac-Asp-Glu-Val-Asp-CHO) or (Ac-DEVD-CHO)		107 (µM) ± 0.027

### In vitro cytotoxicity and anti‑inflammatory activity assessment

**Figure 8:**
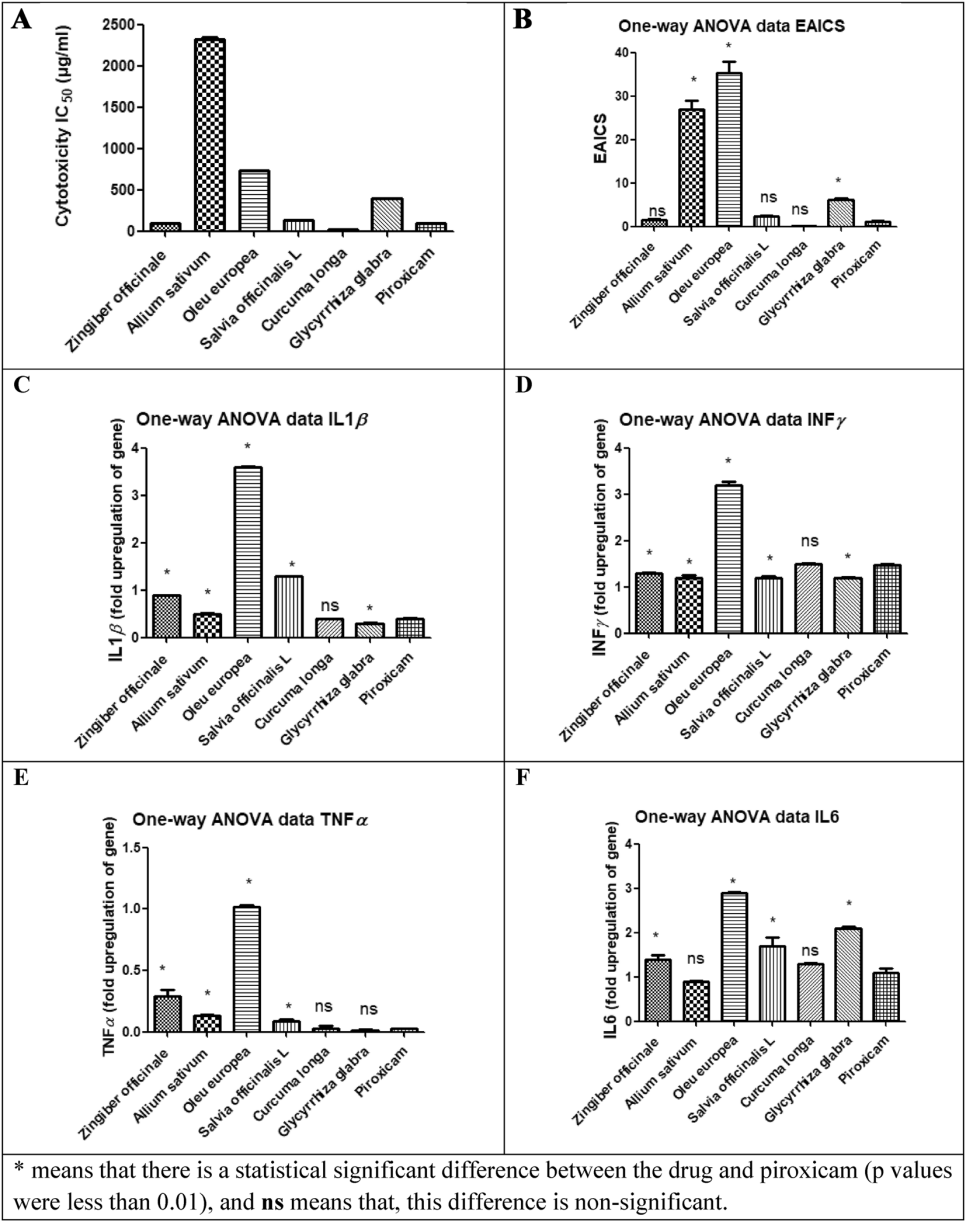
Bar charts illustrating (**A**) cytotoxicity (IC_50_ μg/mL), (**B**) effective anti-inflammatory concentrations (EAICS) (μg/mL) of plant extracts and piroxicam, (**C**) fold upregulation in gene expression of TNF-*α*, (**D**) fold upregulation in gene expression of INF-*γ*, (**E**) fold upregulation in gene expression of IL-1*β* and (**F**) fold upregulation in gene expression of IL-6 by plant extracts and piroxicam.

As unveiled from the prior network pharmacology study, the highest scoring plants, were retrieved to be *Curcuma longa*, *Allium sativum*, *Oleu europea*, *Salvia officinalis L*, *Glycyrrhiza glabra* and *Zingiber officinale*. Despite the fact that network pharmacology is a quick and effective method for anticipating various drug targets in complex diseases, our network analysis results need to be experimentally confirmed by examining the potential bioactivity and correlativity of those plant components in preventing the production of the leading pro-inflammatory mediators that are primarily involved with the different pathways debated above, using these monitoring indices as the basis for a comprehensive evaluation of efficacies. Therefore, to estimate the safety and efficacy of *Allium sativum*, *Glycyrrhiza glabra, Oleu europea*, *Curcuma longa*, *Salvia officinalis L* and *Zingiber officinale* extracts, the cell cytotoxicity 50 (IC_50_), which is the concentration of the drug necessary to reduce the viability of cells by 50%, was established for the extract and the standard anti-inflammatory drug; piroxicam utilizing MTT test. The *Allium sativum*, *Oleu europea*, *Glycyrrhiza glabra* and *Salvia officinalis* extracts demonstrated higher IC_50_ values (2324, 728, 395.9 and 127.1 μg/mL, respectively) than that of piroxicam (100 μg/ mL) illustrating that these extracts are less toxic than piroxicam (Fig. 8A). Then, using WBC activated with lipopolysaccharides (LPS), the anti-inflammatory properties of the studied extracts in comparison to piroxicam were investigated (Fig. 8B). It was inferred that *Curcuma longa*, *Zingiber officinale, Salvia officinalis* and *Glycyrrhiza glabra* extracts (0.19 ± 0.015, 1.57 ± 0.1, 2.31 ± 0.2 and 6.08 ± 0.5 μg/mL, respectively), demonstrated comparable effective anti-inflammatory concentrations (EAICs) with piroxicam (1.21 ± 0.1 μg/mL), indicating the potential anti-inflammatory activities of these extracts. Real-time polymerase chain reaction (PCR) was used to assess the gene expression of four pro-inflammatory mediators (TNF-*α*, IL-1*β*, INF-*γ* and IL-6) in both untreated and lipopolysaccharide (LPS)-treated WBCs in order to ascertain the genetic basis of the anti-inflammatory effect (Fig. 8C–F). LPS increased the expression of the IL-1*β* gene by 6.3 ± 0.1-folds. When *Allium sativum*, *Glycyrrhiza glabra, Oleu europea*, *Curcuma longa*, *Salvia officinalis L* and *Zingiber officinale* were applied to the WBCs, this upregulation was eradicated by 0.5 ± 0.02, 0.3 ± 0.008, 3.6 ± 0.01, 0.4 ± 0.002, 1.3 ± 0.003 and 0.9 ± 0.003-fold, respectively to a degree that was comparable to that employed by piroxicam which exerted 0.4 ± 0.02-fold reduction in gene expression. In terms of INF-*γ*, LPS increased this gene expression by 7.3 ± 0.23-folds. This upregulation was reduced by 1.2 ± 0.05, 1.2 ± 0.03, 1.2 ± 0.009, 1.3 ± 0.01, and 1.5 ± 0.008-fold, respectively, after the WBCs were treated with *Allium sativum*, *Salvia officinalis L, Glycyrrhiza glabra, Zingiber officinale* and *Curcuma longa* extracts, respectively. This reduction was analogous to that caused by piroxicam (1.47 ± 0.03-fold). Meanwhile, LPS induced the TNF-*α* expression by 2.66 ± 0.01-folds that was diminished by the *Glycyrrhiza glabra* and *Curcuma longa* extracts to 0.016 ± 0.001 and 0.02 ± 0.03-folds, respectively. This level was nearly close to what piroxicam had achieved (0.03 ± 0.0003-folds). However, the expression of IL-6 was increased by LPS by 5.4 ± 0.2-folds, and this was reduced to 0.9 ± 0.02 and 1.3 ± 0.01-folds, respectively, by *Allium sativum* and *Curcuma longa* extracts. This amount was very comparable to what piroxicam had accomplished (1.1 ± 0.1-folds). It was noticed that *Curcuma longa* extract showed no statistically significant differences between it and the known synthetic anti-inflammatory drug, piroxicam.

It's interesting to note that both the tested extracts and piroxicam considerably reduced the elevation of the gene expression of IL-1*β*, INF-*γ*, TNF-*α* and IL-6 to a similar degree (error bars were illustrated in Fig. 8 and all experiments p values were less than 0.01). Given the obvious suppression of the increased levels of TNF-*α*, IL-1*β*, INF-*γ*, IL-6 expression, it can be inferred that these studied extracts can serve as a possible natural anti-inflammatory product. These findings were in line with network pharmacology studies, which revealed the tested extracts anti-inflammatory effect to have several targets and pathways.

### Molecular docking studies of hit compounds in the active sites of the highest enriched target genes

The Glide module of the Schrodinger suite software was utilized to calculate the docking XP G scores of the top hit compounds apigenin, luteolin, diallyl trisulfide, silibinin, and allicin, in addition to piroxicam reference, against the active sites of the most enriched immunomodulatory target genes AKT1, CASP3, PTGS2, NOS3 and TP53. From Table 5, it can be noticed that silibinin had the smallest XP G score against RAC-alpha serine/threonine-protein kinase (AKT1) followed by caspase-3 and finally tumor suppressor protein TP53 with binding energies of − 11.725, − 8.855 and − 7.13 kcal/mol, respectively. Whereas luteolin and apigenin exhibited the highest stabilized interactions with RAC-alpha serine/threonine-protein kinase with binding energies of − 10.012 and − 9.013 kcal/mol, respectively followed by prostaglandin-endoperoxide synthase 2 with binding energies of − 7.534 and − 6.811 kcal/mol, respectively and then tumor suppressor protein TP53 with binding energies of − 6.732 and − 6.685 kcal/mol, respectively.

**Table 5:** XP G scores of the top hit compounds in the C-T network versus the highest enriched target genes.

	RAC-alpha serine/threonine-protein kinase (3O96)	Caspase-3 (3DEI)	Tumor suppressor protein TP53 (2VUK)	Prostaglandin-endoperoxide synthase 2 (5F1A)	Endothelial nitric oxide synthase (1M9J)
Luteolin	− 10.012	− 6.543	− 6.732	− 7.534	− 6.46
Apigenin	− 9.013	− 6.245	− 6.685	− 6.811	− 5.947
Allicin	− 3.335	− 2.313	− 3.105	− 4.509	1.456
Diallyl trisulfide	− 1.607	− 1.439	− 1.535	− 3.363	2.36
Silibinin	− 11.725	− 8.855	− 7.13	–	–
Piroxicam	− 3.664	− 4.176	− 0.956	− 3.868	1.524

**Figure 9:**
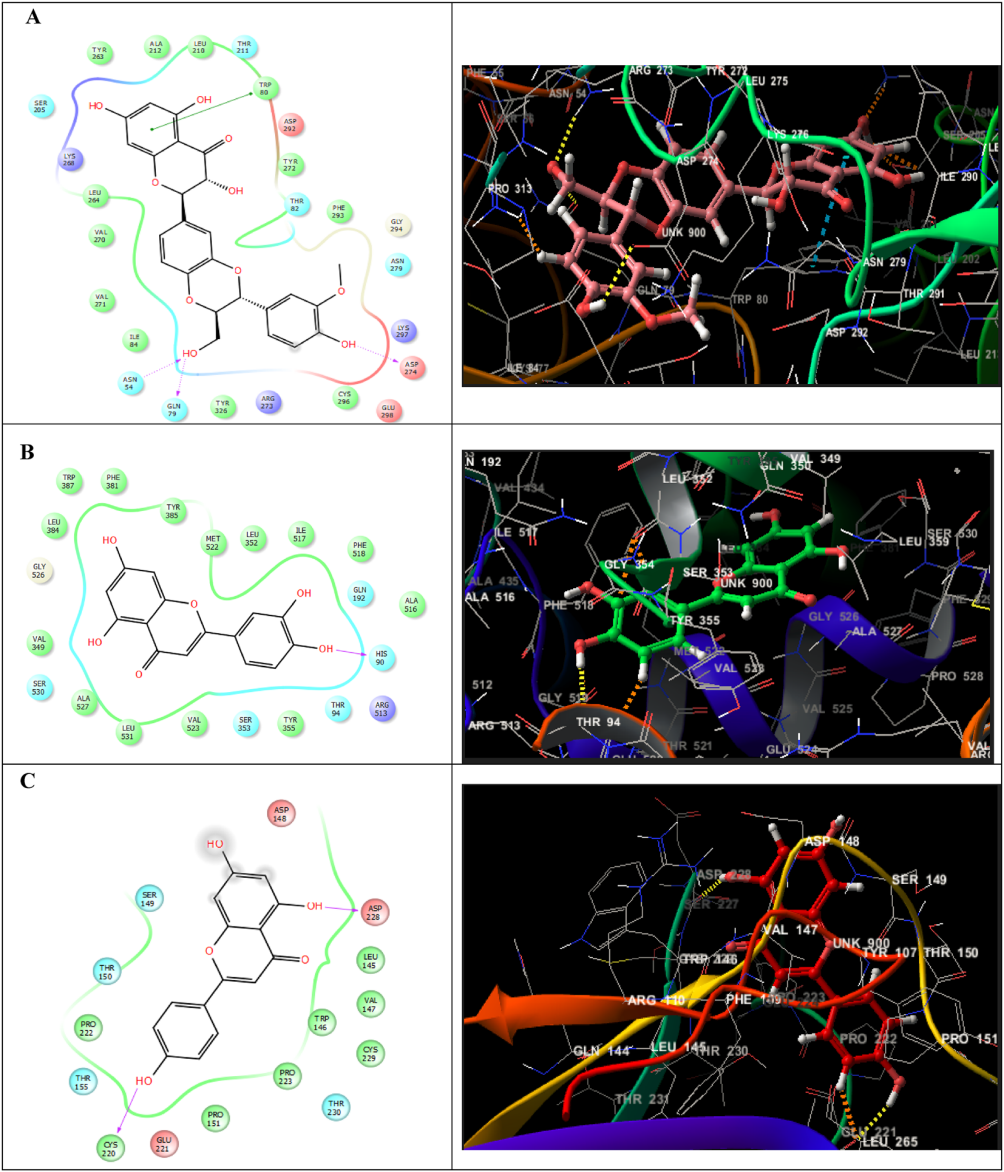
2D and 3D interaction diagrams of: (**A**) Silibinin in the active site of RAC-alpha serine/threonine-protein kinase (PBD ID 3O96) (**B**) Luteolin in the active site of prostaglandin endoperoxide synthase 2 (PDB ID 5F1A) and (**C**) Apigenin in the active site of tumor suppressor protein TP53 (PDB ID 2VUK).

Based on the two- and three-dimensional interaction diagrams illustrated in Fig. 9A, it was identified that silibinin is well integrated in the active site of AKT1 (PBD ID 3O96) via constituting hydrogen bonds with Asn54, Gln79 and Asp274, and a pi–pi stacking interaction with Trp80 as well as positively charged interactions with Arg273, Lys297 and Lys268. Moreover, negatively charged interactions with Asp274, Asp292 and Glu298 were noticed. Also, hydrophobic interactions with Val270, Val271, Leu210, Leu264, Tyr263, Tyr126, Tyr272, Phe293, Ile84, Cys296 and Trp80 were observed. Silibinin, additionally tied to the core protein; AKT1 throughout polar interactions with the amino acid residues: Asn54, Asn279, Gln79, Thr82, Thr211 and Ser205. Similarly, luteolin and apigenin demonstrated good molecular binding capability to AKT1 through numerous connection sites.

On the other hand, the interaction of luteolin with prostaglandin-endoperoxide synthase 2 (PTGS2) (PDB ID 5F1A) included one hydrogen bond between the 4' hydroxyl group and His90 residue. Along with these interactions, there were polar interactions with His90, Gln192, Ser530, Ser353 and Thr94 as well as hydrophobic interactions with Leu384, Lue531, Leu352, Phe518, Phe381, Met522, Ile517, Ala527, Ala516, Trp387, Val349, Val523, Tyr355, Tyr385. A charged positive interaction with Arg513 was also detected (Fig. 9B).

While the majority of interactions with the tumor suppressor protein TP53 (PDB ID 2VUK), in which apigenin was particularly integrated, were hydrophobic. Pro151, Pro223, Pro222, Cys220, Cys229, Trp146, Val147 and Leu145 were all found to interact hydrophobically with apigenin, and the 5, 4'-hydroxyl groups were particularly integrated in two hydrogen bonds with Cys220 and Asp228. This may have a significant impact on apigenin increased stability in the binding pocket of the target protein. Polar interactions with Thr150, Thr155, Thr230, and Ser149 were also present, in addition to charged negative interactions with Glu221, Asp228 and Asp148 (Fig. 9C). All of these investigations could successfully confirm the outcomes indicated above, suggesting the feasibility of this integrated strategy and serving as a guide to systematically uncover the therapeutic mechanisms of these naturally occurring plant components for the treatment of inflammation.

### Molecular dynamics simulations stability test for hit protein–ligand complexes

**Figure 10:**
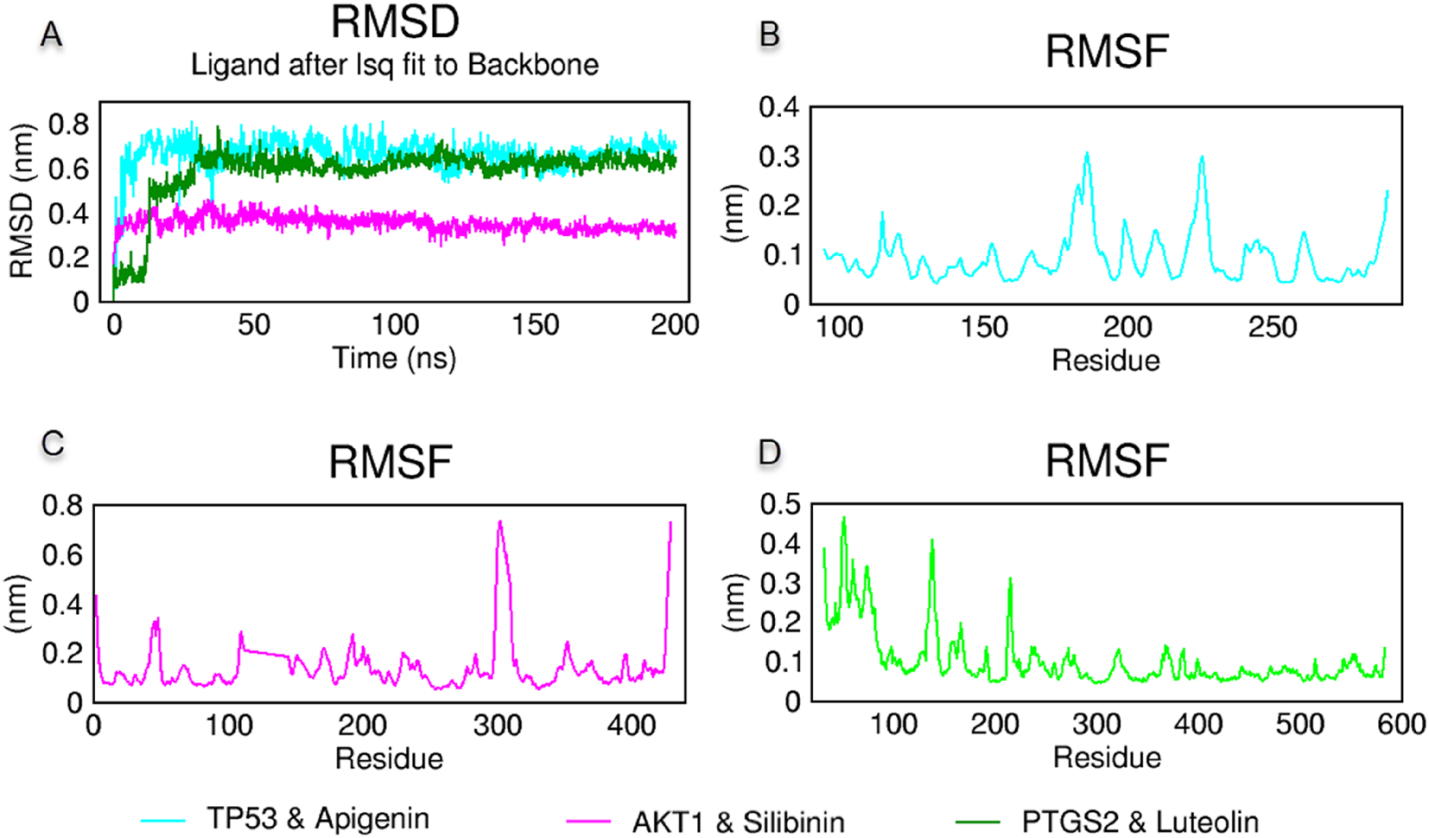
Molecular dynamics simulations trajectory analysis of TP53 & apigenin, AKT1 & silibinin, and PTGS2 & luteolin complexes for 200 ns. (**A**) The root mean square deviation (RMSD) for for all three complexes, (**B**) The root mean square fluctuation (RMSF) of TP53 & apigenin, (**C**) AKT1 & silibinin, and (**D**) PTGS2 & luteolin protein–ligand complexes during the molecular dynamics simulation.

Two hundred (200) ns duration molecular dynamics (MD) simulation was performed to analyze the in silico stability of TP53 & apigenin, AKT1 & silibinin, and PTGS2 & luteolin protein–ligand complexes obtained from the molecular docking study^70^. The root mean square deviation (RMSD) measurements were performed for the shifts of ligands with respect to residues at the binding sites, and root mean square fluctuation (RMSF) measurements were made from the MD trajectory to explain fluctuations in protein structure^71^. As given in Fig. 10A, all three protein–ligand complexes stabilized and remained stable after the first 20 ns. TP53 & apigenin and PTGS2 & luteolin are stable around 0.7 nm and AKT1 & silibinin complex is around 0.4 nm. Further trajectory analysis of RMSF gave fluctuations below 0.3 nm for TP53 & apigenin complex, AKT1 & silibinin complex below 0.8 nm, and PTGS2 & luteolin complex below 0.5 nm, as given in Fig. 10B–D, respectively.

**Figure 11:**
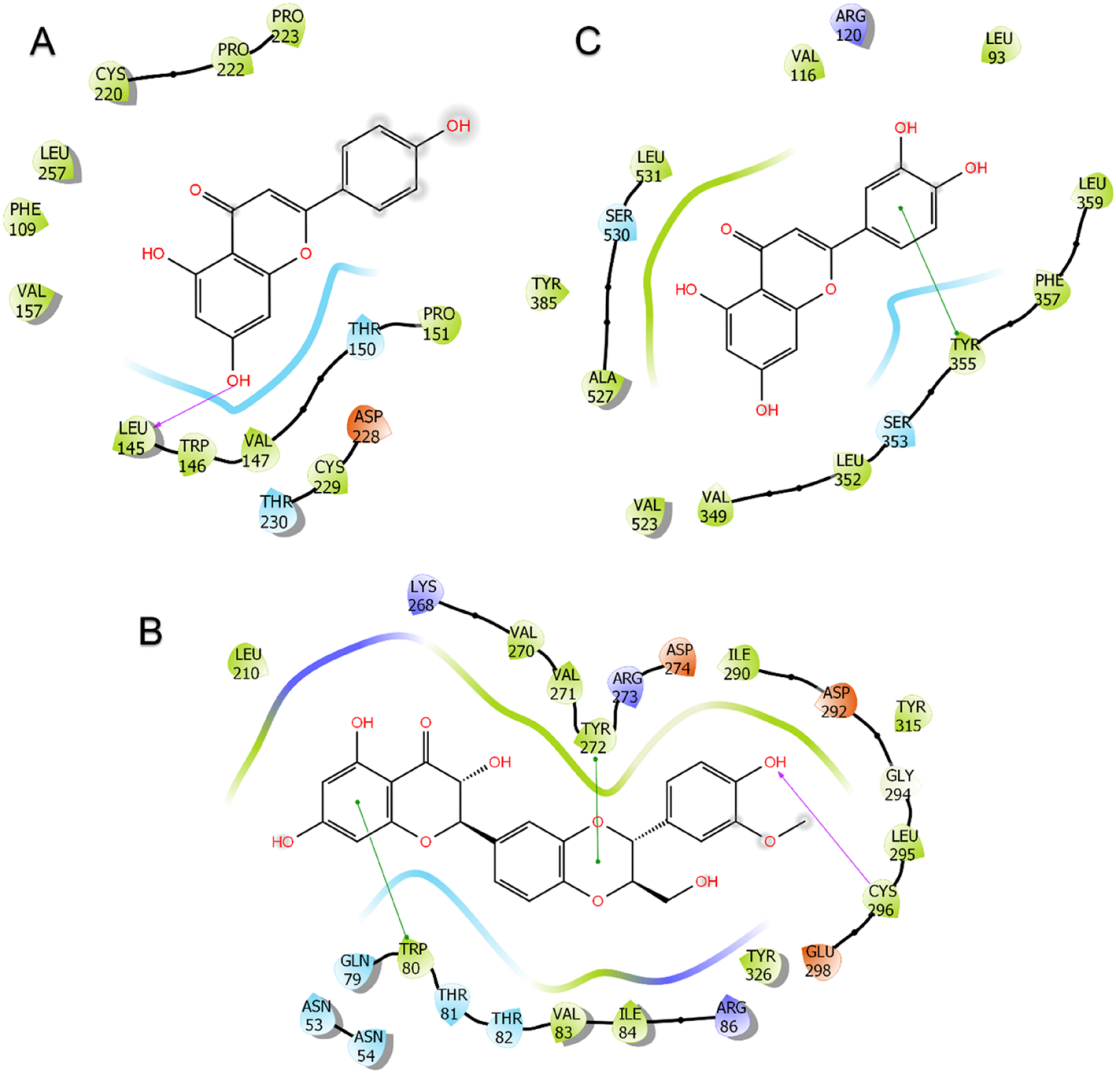
Protein–ligand interactions end of 200 ns from molecular dynamics simulation of (**A**) TP53 & apigenin, (**B**) AKT1 & silibinin, and (**C**) PTGS2 & luteolin protein–ligand complexes.

Schematic 2D diagrams of protein–ligand interactions at 200 ns are given in Fig. 11 to demonstrate protein–ligand stabilities^72^. As shown in Fig. 11A, apigenin maintains its stability by forming an H bond with Leu145 at the active site of TP53, a negative charge with Asp228, and hydrophobic interactions with Cys220. As given in Fig. 11B, silibinin remained stable in the AKT1 active pocket, as in the molecular docking pose, giving Pi–Pi stacking interaction with Trp80, as well as an H bond interaction with Thr272 with Pi–Pi stacking and Cys296. As given in Fig. 11C, luteolin gave Pi–Pi stacking interactions mainly with Thr355 at the PTGS2 active site and maintained its stability. Finally, animation videos from MD trajectory were created and provided in supplementary materials (Videos S1–S3) to monitor protein–ligand interactions full-time and better explain ligand stability. Analysis of MD simulations revealed that all three protein–ligand complexes are quite stable.

## Experimental

**Figure 12:**
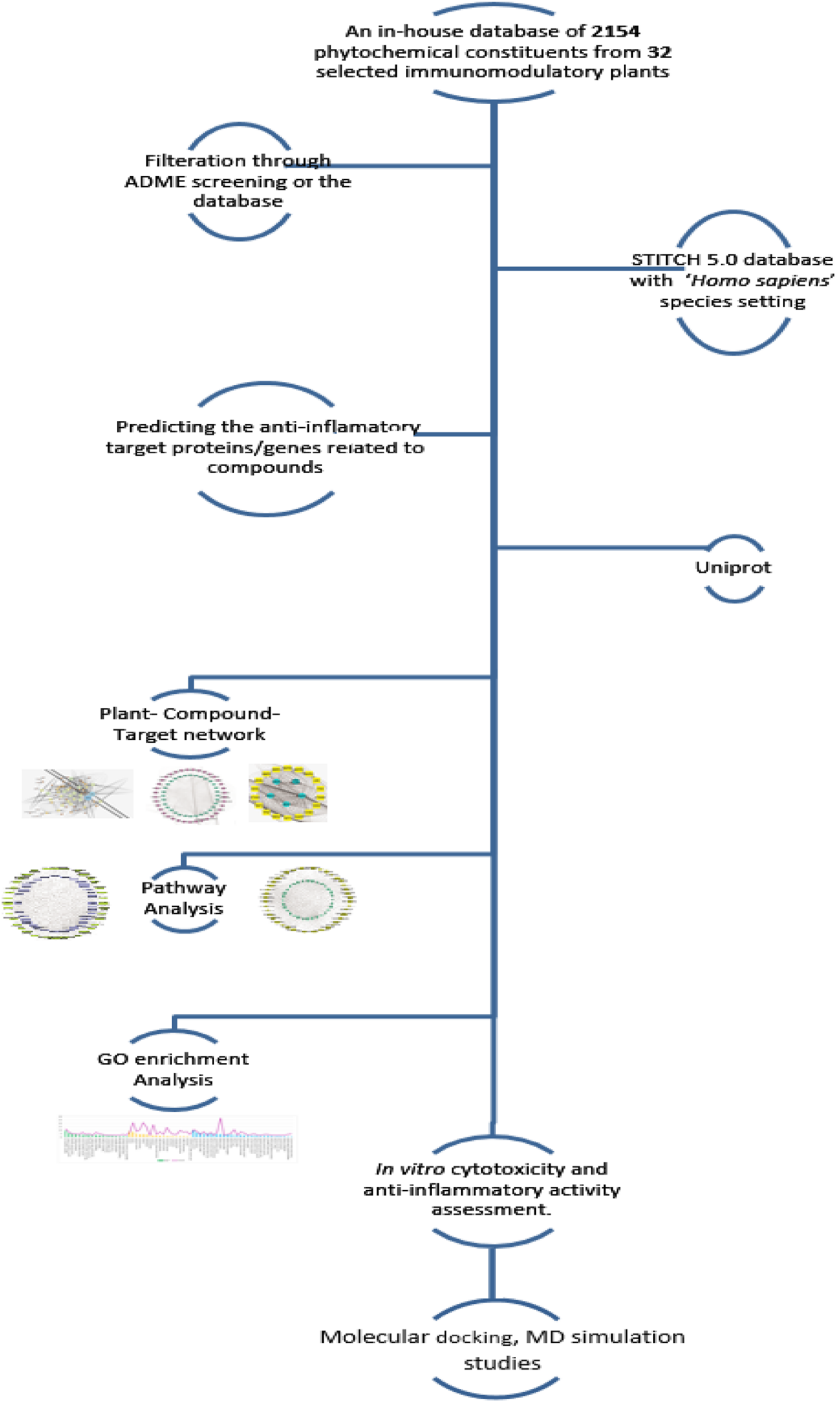
Flow chart for the whole analysis implemented in the study.

The study design is summarized as shown in Fig. 12.

### Assembling of an in-house database

Depending on our previous study of the chemical makeup of 32 selected immunomodulatory plants, 2154 phytochemical constituents were obtained for compilation of an in-house database as illustrated in Table S3. These plants were selected according to their traditionally known action to treat immune related disorders or based on prior in vivo, in vitro or clinical trials from literature review as mentioned in Khairy et al.^73^ and summarized briefly in Table 6. Different resources including Dictionary of natural products (http://dnp.chemnetbase.com/faces/chemical/ChemicalSearch.xhtml;jsessionid=0A16BF52515E734B15A96DCBAE7788B9), National Centre for Biotechnology Information's PubChem database (https://pubchem.ncbi.nlm.nih.gov/), ChEMBL (https://www.ebi.ac.uk/chembl/) and Chemdraw software (CambridgeSoft Corporation, Cambridge, USA) were used to provide 2D structures of these compounds in (.sdf) format. Schrodinger software (2017A) was utilized to transform these 2D structures to SMILES format.

**Table 6:** The chosen immunomodulatory plants identified either traditionally or based on their prior literature review studies, to heal immune related diseases.

Plant name	Family	Pharmacological action	References
*Allium sativum*	Amaryllidaceae	Prevents the pro-inflammatory cytokines IL-6 and monocyte chemoattractant protein-1 (MCP-1)	^74^
*Artemisia annua*	Asteraceae	Suppresses the delayed-type hypersensitivity reaction—Had inhibitory effect on calmodulin	^75,76^
*Calendula officinalis*	Asteraceae	Possess anti-inflammatory activities—inhibits the mitogen-induced lymphocyte proliferation	^77^
*Camellia sinensis*	Theaceae	Possess anti-inflammatory properties	^74^
*Cannabis sativa*	Cannabaceae	Inhibits the proliferation of lymphocytes	^78^
*Cichorium intybus*	Asteraceae	Inhibits the lymphocyte proliferation assay in the presence of PHA (phytohemagglutinin)	^77^
*Citrullus colocynthis*	Cucurbitaceae	Reduces the cell proliferation induced by concanavalin A (con-A)	^79^
*Citrus aurantium*	Rutaceae	Active against concanavalin A and LPS (lipopolysaccharide) induced proliferation in both thymocytes and splenocytes, respectively	^80^
*Curcuma longa*	Zingiberaceae	Potent suppressor to various pro-inflammatory cytokines such as TNF-*α*, IL-6, IL-8, IL-12 and IL-1*β*—It also suppresses TH1 and TH17 -mediated inflammatory response	^81,82^
*Cynara scolymus*	Asteraceae	High doses suppress the delayed-type hypersensitivity response	^83^
*Ephedra sinica*	Ephedraceae	Inhibits the splenocyte proliferation and suppresses the humoral immune response	^84^
*Euphorbia species ((E. royleana, E. lacteal, E. tirucalli Boiss))*	Euphorbiaceae	Reduces CD4 + T cells and neutrophils, inhibits IL-2 and the process of phagocytosis	^75,85^
*Glycyrrhiza glabra*	Fabaceae	Down-regulates IgE production, inhibits calcineurin activity and T cell proliferation	^75,86^
*Lawsonia inermis*	Lythraceae	Inhibits lymphocyte transformation	^75,87^
*Linum usitatissimum*	Linaceae	Inhibits mitogen (concanavalin A)-induced response of human peripheral blood lymphocytes	^88^
*Matricaria chamomilla*	Asteraceae	Disrupts Th1/Th2 balance to Th1 upregulation and decreases the IL-6 and TNF-*α* production	^89,90^
*Nigella sativa*	Ranunculaceae	Reduces the serum antibody titre, dysregulates the intracellular killing and cytokine production	^91^
*Oleu europea*	Oleaceae	Modulates the production of pro-inflammatory mediators, reduces the expression of COX2 and causes a dose-dependent reduction of PGE2	^92^
*Panax ginseng*	Araliaceae	Inhibits Th1 and helper T cell 17 (Th17) differentiation—Decreases pro-inflammatory cytokines	^93^
*Physalis peruviana*	Solanaceae	Reduces the release of IL-6, IL-8, and monocyte chemoattractant protein-1 (MCP-1)	^94^
*Piper cubeba*	Piperaceae	Reduces the cell proliferation induced by concanavalin A	^95^
*Propolis*	Bee product	High doses inhibit lymphocyte proliferation and possesses anti-inflammatory properties	^96^
*Punica granatum*	Lythraceae	Inhibits the activation of the nuclear factor of activated T cells, decreases CD3 + T cell infiltration of the inflamed tissue	^75,97^
*Salvia officinalis L*	Lamiaceae	Decreases the inflammation induced by LPS and reduces the blood levels of TNF-*α* and NF-*κ*B	^98^
*Sesamum indicum*	Pedaliaceae	Decreases white blood cell count, erythrocyte sedimentation rate, IL-6 and TNF-*α*	^99^
*Silybum marianum*	Asteraceae	Silymarin reduces Th1-linked cytokines (IL-2, IFN-*γ*, TNF-*α*) in addition to reduction of MAPKs’ activities (ERK1/2 and P38)	^100^
*Tanacetum parthenium*	Asteraceae	Reduces several pro-inflammatory enzymes and mediators including phosphodiesterase-3 and 4, 5-lipoxygenase, NO, PGE2, IL-4 and TNF-*α*	^101^
*Trigonella foenum-graecum*	Fabaceae	Inhibits the inflammatory enzymes such as cyclooxygenase and lipoxygenase Reduces the levels of TNF-*α* , IL-6, ↓arthritic index and ↓rheumatoid factor	^102^
*Urtica dioica*	Urticaceae	Suppresses the human dendritic cells maturation, ↓ induction of primary T cell responses and ↓ TNF-*α*	^103^
*Withania somnifera*	Solanaceae	Produces immunosuppressive action on B and T cell activity in hyper-immune states	^74^
*Xanthium strumarium L*	Asteraceae	Suppresses the overproduction of IL-1*β*, TNF-*α*, cyclooxygenase -2 and 5- lipoxygenase	^104^
*Zingiber officinale*	Zingiberaceae	Reduces inflammation in arthritis by inhibiting cyclooxygenase and lipoxygenase pathways	^74^

### ADME and drug-likeness filtration

Using the Qikprop software (Schrodinger suite 2017A), plant constituents obtained from the database were filtered out through the application of ADME system and Lipinski's rule of five^28^. In order to determine if the examined compounds would be good therapeutic candidates, a variety of physio-chemical attributes were estimated. In this consider, compounds with an anticipated oral bioavailability (OB) ≤ 30 were not included in this investigation. In expansion, compounds that met fewer than three of Lipinski's five requirements were also disregarded.

### Network pharmacology-based analysis

#### Target genes related to the filtered constituents

The target genes associated with the chosen chemical constituents were identified utilizing STITCH DB (http://stitch.embl.de/, ver. 5.0) using the *'Homo sapiens'* species setting^25^. Genes information like genes ID, names, organism and function were obtained from UniProt (http:// www.uniprot.org/)^25,26^. Only the *‘Homo sapiens’* proteins connected to immunosuppressive disorders were kept. Then, using STRING database (https://string-db.org)^36^, the protein–protein interaction network (PPI network) was created.

### Networks construction and pathway analyses

For further investigation of the multi-level mechanisms of action of plant components in the treatment of immunosuppressive disorders, different types of networks including plant-constituent, gene-pathway, constituent-target gene and constituent-gene-pathway networks—were built using Cytoscape 3.8.2 (http://www.cytoscape.org/). The nodes in these networks stand for genes, constituents and pathways, while the edges denote the interactions existing between them. The Cytoscape network analyzer plug-in was used to compute the network parameters. The importance of nodes in each built network was evaluated utilizing Cytoscape combined score of interactions.

### Gene ontology (GO) enrichment analysis of the identified targets

The Kyoto Encyclopedia of Genes and Genomes (KEGG) pathways and the Database for Annotation, Visualization and Integrated Discovery (DAVID) ver. 6.8 (https://david.ncifcrf.gov/) were searched to learn more about gene ontology and to identify the canonical pathways, cellular components, biological processes and molecular functions that were closely related to the target genes^36,105^. The only selected pathways had *P*-values ≤ 0.05. *P* ≤ 0.05 was considered to be statistically significant.

### Evaluation of network pharmacology analysis

The network pharmacology evaluation is conducted from three aspects—reliability, standardization, and rationality. Taking in consideration that the network pharmacology evaluation process includes both general evaluation and scalability evaluation. The evaluation process was carried out according to the method described previously by Li^68^.

### Preparation of the extracts test solutions

According to the results of the network pharmacology study, the best-scoring plants (*Curcuma longa*, *Allium sativum*, *Oleu europea*, *Salvia officinalis L*, *Glycyrrhiza glabra* and *Zingiber officinale*), were bought, from a reputable local market in Alexandria, Egypt. Professor Sania Ahmad, a professor in Faculty of Science at Alexandria University examined the tested samples macroscopically and microscopically in order to be able to authenticate them. Voucher specimens were registered in the Pharmacognosy Department herbarium, Faculty of Pharmacy, Alexandria University under the following codes: (CL 005, AS 2021, OE 007, SO 001, GG 002 and ZO 009), respectively. Our study complies with relevant institutional, national, and international guidelines and legislation. The plants were firstly air dried before being individually double macerated on 70% ethanol for extraction. The obtained extracts were filtered and then evaporated using a rotary evaporator (BuchiRotavapor Model R-200, Flawil, Switzerland), at reduced pressure. To make a stock solution from each sample, 1 mg of each tested extract was added to a separate 10 mL volumetric flask, dissolved in 1 mL of dimethyl sulfoxide (DMSO), and finally the volume was completed to 10 mL using distilled water. The assay buffer solution (100 mM Tris and 0.1% Triton X-100, pH 8.0) was then used to dilute the stock solutions of each sample under examination to produce different concentrations of each sample.

### Experimental verification of top target proteins

**RAC-alpha serine/threonine-protein kinase (Akt1) inhibitory activity using spectrophotometric assay.**

The Akt1 inhibitory activity of the top four plants resulted from network pharmacology analysis was measured using a colorimetric assay of released adenosine diphosphate (ADP)^106^. The ADP assay kit offers a straightforward method for detecting ADP in a wide range of samples, specifically those containing reducing agents that may interfere with oxidase-based assays. ADP concentration was evaluated using a coupled enzyme colorimetric assay (450 nm) that was proportional to the quantity of ADP present. The bioassays were performed as described by Al-Sha’er et al.^107^ and mentioned in supplementary material. Saturosporine, an Akt inhibitor, was tested as a positive control, while the negative controls were prepared by adding the substrate after terminating the reaction.

### Caspase-3 inhibitory activity assay

The inhibitory activity of the top four plants resulted from network pharmacology analysis on caspase 3 gene was quantified as illustrated on caspase 3 colorimetric assay kit (CASP-3-C)^108^ supplied by Sigma, and described by Ros et al.^109^ as in supplementary materials.

### Assessment of the cytotoxicity and anti-inflammatory activity

The MTT test was used to evaluate the cytotoxicity of the various extracts in comparison to piroxicam. Each extract's effective anti-inflammatory concentrations (EAICs) were calculated in a culture of lipopolysaccharides (LPS) activated human WBCs obtained from human volunteers with ethical approval from ethical committee (IRB NO: 00012098). Expression levels of IL-6, IL-1*β*, TNF-*α* and INF-*γ* were determined using real-time polymerase chain reaction (PCR). The means ± standard deviations of three distinct replicates were used to express the results. The method described by Darwish et al.^110,111^, as given in the supplementary data, contains the specific details about the procedures. Statistical analysis of the results was performed using One-way ANOVA test- Tukey post-test options implemented on GraphPad Prism 5 program.

### Molecular docking studies

Crystallographic structure of the most enriched target genes (AKT1, CASP3, PTGS2, NOS3 and TP53), identified through network pharmacology analysis, were obtained from the Protein Data Bank (PDB). Each protein crystal structure was chosen based on the highest resolution possible. Through the use of the protein preparation wizard (OPLS 3 force field) module run in the Schrodinger suit, the preparation and energy minimization of crystal structures of the target proteins were carried out. The protein optimization was followed by the assignment of hydrogen bonds and bond order. At pH 7, zero order bonds to metals and disulphide bonds were also constructed. Next, all water molecules that were farther than 5°A away from the active site were eliminated. The grid boxes were constructed using the residues implicated in interactions with the co-crystallized ligands. The compounds 2D structures were loaded as (.sdf) format into the Lig Prep 2.3 module (Lig Prep, version 2.3, 2015, Schrödinger, USA) to build the least energy 3D structure for each compound and search for alternate conformers. The ionisation states were adjusted to create all conceivable states at pH 7. Molecular docking analyses were performed utilizing the Glide docking program of Maestro molecular modelling package executing extra-precision (XP-Glide) module. The ligand–target interactions such as hydrogen bond, hydrophobic interactions, ion pair interactions together with the binding modes of the identified compounds were demonstrated using Maestro interface.

### Molecular dynamics simulations

To test the stability of protein–ligand complexes derived from molecular docking studies with Glide, MD simulations were run with Gromacs v2020.1^112^. The CHARMM-GUI server was used to prepare the necessary input files^113^. Using AMBER99SB force fields, proteins and compounds’ topology files were created^114^. The TIP3 water model was used to dissolve the protein–ligand complexes, using a rectangular box type 10 Å away from the complexes. Then, 0.15 KCl salt was added to neutralize the solution. The created system was minimized to 5000 nsteps with the steep integrator. Nose–Hoover and Parrinello-Rahman algorithms were used to equilibrate it using NVT/NPT ensemble steps of 0.3 ns duration at 300 K and 1 atm pressure. 200 ns MD simulation was run and 1000 frame was recorded. The root mean square deviation (RMSD) and the root mean square fluctuation (RMSF) were measured with gmx scripts^115,116^. RMSD and RMSF plots were created with QtGrace v2.0.6. The PyMol Molecular Graphics System version 2.5.2 software was used to make MD trajectory videos and protein–ligand binding poses visualization.

## Conclusion

Given that medicinal plants are multi-component and the network pharmacology approach emphasizes the idea of “network target, multicomponent therapeutics,” in a holistic manner analogous to the complex matrices of medicinal herbals, this strategy is thought to be appropriate for understanding the mechanism of action of medicinal plants. In this study, network pharmacology-based analysis of 2154 phytochemical constituents obtained from 32 selected immunomodulatory plants was carried out. A total of 37 constituents and 32 targets were found to be involved in 40 immunomodulatory-associated pathways. Apigenin, luteolin, diallyl trisulfide, silibinin and allicin had the highest percentage of C-T interactions, where the target genes AKT1, CASP3, PTGS2, NOS3, TP53 and MMP9 were found to be the most enriched ones by possessing the highest combined scores, suggesting that they may be the key nodes in C-T network. KEGG analysis illustrated that pathways in cancer, fluid shear stress and atherosclerosis, relaxin signaling pathway, IL-17 signaling pathway and FoxO signaling pathway had the largest number of observed genes and the smallest false discovery rate. Additionally, a combined plant-constituent-target-pathway network demonstrated that *Curcuma longa*, *Allium sativum*, *Oleu europea, Salvia officinalis L*, *Glycyrrhiza glabra* and *Silybum marianum* had the highest number of P-C-T-P interactions which would suggest that these plants have more active substances that can act as immunomodulatory agents. However, the main drawback in the current network pharmacology analysis studies is the insufficient scientific verification as it needs extensive *in-vitro*, *in-vivo* and clinical investigations^68^.

Furthermore, molecular docking analysis of the top hit compounds; apigenin, luteolin, diallyl trisulfide, silibinin, and allicin, against the active sites of the most enriched immunomodulatory target genes; AKT1, CASP3, PTGS2, NOS3 and TP53 was carried out. The results revealed that silibinin had the lowest binding energy with RAC-alpha serine/threonine-protein kinase (AKT1) followed by caspase-3 and finally tumor suppressor protein TP53, whereas luteolin and apigenin exhibited the highest stabilized interactions with RAC-alpha serine/threonine-protein kinase followed by prostaglandin-endoperoxide synthase 2 and then tumor suppressor protein TP53. The extracts of the highest scoring plants retrieved from network analysis, were then subjected to in vitro anti-inflammatory and cytotoxicity testing exhibiting outcomes that are equivalent to those of piroxicam. These plants are suggested as prospective sources of immunomodulatory agents by this study, which also offers a thorough explanation of the mechanism underlying the extracts’ supposed anti-inflammatory action. To support our findings, additional in vivo and clinical research are advised.

## Supplementary Information

Supplementary Information 1.

Supplementary Information 2.

Supplementary Information 3.

Supplementary Information 4.

Supplementary Information 5.

## Data Availability

All data generated or analyzed during this study are included in this article (and its supplementary information files).
