# Supplementary material for: Forecasting of potential anti-inflammatory targets of some immunomodulatory plants and their constituents using in vitro, molecular docking and network pharmacology-based analysis
Source: Sci Rep. 2023 Jun 12;13:9539. doi: 10.1038/s41598-023-36540-3 (PMC10260966; doi:10.1038/s41598-023-36540-3)
Supplement: Supplementary file 1 — Supplementary Information 1. [file 41598_2023_36540_MOESM1_ESM.docx]

**Supplementary materials**

**Forecasting of potential anti-inflammatory targets of some immunomodulatory plants and their constituents using *in vitro*, molecular docking and network pharmacology-based analysis**

**Supplementary tables**

**Table S1: Results of ADME screening of the database of immunomodulatory plants (rule of five and percent human oral absorption).**

**Table S2: The distribution % of the compound-target interactions on the chosen plant constituents.**

**Table S3: An in-house database of 2154 constituents from 32 selected immunomodulatory plants.**

**Video S1. MD Simulation animation of 200 snapshots between 0 and 200 ns of TP53 and Apigenin complex.**

**Video S2. MD Simulation animation of 200 snapshots between 0 and 200 ns of AKT1 and Silibinin complex.**

**Video S3. MD Simulation animation of 200 snapshots between 0 and 200 ns of PTGS2 and Luteolin complex.**

# Experimental

**Quantification of Akt1 inhibitory activity using a spectrophotometric assay**

The ATPase activity of Akt1 was quantified using a colorimetric measurement of the released ADP [^1^](#_ENREF_1). The ADP assay kit provides a simple and direct procedure for measuring the ADP in a variety of samples, particularly those containing reducing agents that may interfere with oxidase-based assays. ADP concentration was determined by a coupled enzyme colorimetric assay (450 nm) proportional to the amount of ADP present. The bioassays were performed as follows: in a 96-well clear plate, the reaction mixtures were prepared to get a final volume of 25 µL that contains: 5 µL (50 ng/well) of the human Akt1 enzyme, 5 µL of one of the tested compounds (100 µM), 5 µL of ATP solution (100 µM), 5 µL of the Akt substrate (100 ng/well), and 5 µL of the assay buffer. The plate was sealed and the reaction was incubated at 37 ◦C for 2 h. The reaction was stopped by the addition of 25 µL of ADP colorimetric kit [^1^](#_ENREF_1), left for 30 min in dark and the absorbance was measured at 450 nm using a plate reader (Bio-Tek instruments ELx 800, Winooski, VT). The calibration curve was prepared using 5 different concentrations of ADP (10–200 µM). The final concentration of DMSO did not exceed 1.0%. Akt1 inhibition was calculated as a percentage of activity compared to the uninhibited control. Akt standard inhibitor was tested as a positive control, while the negative controls were prepared by adding the substrate after terminating the reaction.

**Caspase 3 inhibitory activity assay**

# Caspase 3 activity within the plant extract samples was measured using the Caspase 3 Assay Kit, Colorimetric (Sigma Aldrich, Australia). This assay is based on the monitoring of DEVD-p-nitroanilide cleavage in yellow-colored p-nitroaniline (pNA) through time [^2^](#_ENREF_2). For each assay, in addition to the plant extract samples tested, the following standards were undertaken: (i) a blank which consisted of assay buffer and substrate only, in order to verify that the coloration was not due to substrate degradation through time, (ii) a negative control for each sample containing a caspase 3 inhibitor to determine the signal baseline, and (iii) a positive control containing commercial caspase 3 provided with the kit, allowing the verification of the test efficiency. All samples and standards were loaded as quadruplicates. The temperature was maintained at 37∘ C during the assay and the kinetics of the reaction were followed for 3 h by measuring absorbance at 405 nm every minute on each well using the plate reader software, directly after addition of the caspase 3 substrate. To convert the absorbance values to the concentration of substrate degraded, a standard curve was produced relating the concentration of pNA with the obtained absorbance values. The reagents were added to the wells to a final volume of 100 𝜇L.

# Cytotoxicity and anti-inflammatory activity test (MTT assay)

#

# Isolation and cultivation of human white blood cells

**Reagents**

- Ammonium chloride lysis solution (10X concentration) composed of NH_4_Cl 8.02gm, NaHCO_3_ 0.84gm and EDTA 0.37gm.
- Working solution of ammonium chloride (100 ml) composed of (10ml of above solution with 90 ml distilled water).
- RPMI media containing 10% fetal bovine and 2% L- glutamine.
- Phosphate buffer saline (137 mM NaCl, 2.7 mM KCL, 10 mM Na_2_HPO_4_ and 10 mM KH_2_PO_4_) pH 7.4.
- Trypan blue dye 0.5%.

**Procedure**

A whole blood specimen from human volunteers was obtained in a sterile heparin tube and an aliquot about 1ml blood was taken into 15ml centrifuge tube then the tube was filled to capacity with fresh cold lysing solution then inverted for ~10 minutes at room temperature until the liquid became clear red. Specimens were centrifuged at 4^o^ C for 10 min at 2000 rpm, supernatant was decanted and the tubes were allowed to drain, The pellets (WBCs) were suspended in 10 ml cold PBS, recentrifuged and pellets were resuspended in RPMI culture medium. WBCs viability and counting were assessed using the dye exclusion method of (Louis and Siegel; 2011). A portion of the cell suspension (e.g., 50 µl) was mixed with an equal volume of 0.5% trypan blue staining solution and loaded onto hemocytometer. Both viable "unstained" and nonviable "stained" cells were counted in each of the four corner quadrants (A, B, C, D).

**Calculation**

**N / ml = mean of WBCs counting x10^4^ × D**

N: Number of viable or nonviable cells

D: Sample dilution (1:1 with the trypan blue).

**% of cell viability = Number of viable cells × 100**

**Total number of cells**

At least 90% of the cells must be viable in order to use the cells for assays, Then WBCs that were cultured in RPMI media, incubated in CO_2_ incubator for six days. WBCs were seeded as 100,000 cells/ well (96 well cell culture plate) and incubated in CO_2_ incubator (37°C, 5% CO_2_, and 90% relative humidity).

##

## Assessment of cytotoxicity of the crude extracts compared to piroxicam

**Reagents**

- Reference anti-inflammatory drugs: piroxicam serial dilution (3.125, 6.25, 12.5, 25 and 50 µg/ml in culture media)
- MTT solution (5 mg/ml in PBS)
- Dimethylsulfoxide (DMSO)
- Culture media (RPMI containing 10% fetal bovine and 2% L- glutamine)

**Procedure**

In this assay, A volume of 200 µl of cultured medium that contained 100,000 WBCs / well (96-well cell culture plate) were plated with different concentrations (0, 3.125, 6.25, 12.5, 25 and 50 μg/ml) of crude extracts in RPMI medium without fetal bovine serum or standard anti-inflammatory drugs piroxicam then the plate was incubated for 72 h in CO_2_ incubator (37°C, 5% CO_2_, and 90% relative humidity). After 72 h of incubation, a volume of 20μl of MTT solution was added to each well and plates were incubated for 3 h in CO_2_ incubator to allow the MTT to be reacted. After incubation, the plates were centrifuged at 1650 rpm for 10 min and the medium was discarded. The formazan crystals (MTT byproduct) were re-suspended in 100μl DMSO and reading was measured at a wavelength of 570 nm using optima spectrophotometer for detecting safe dose, which cause 100% cell viability.

**The % viability was calculated as follow: (A_T_-A_b_ /A_C_-A_b_) x 100**

**A_T_** = mean absorbances of cells treated with different concentration of each plant extract

**A_C_** = mean absorbances of control untreated cells with culture medium only

**A_b_**_=_ mean absorbances of cells treated with vehicle of plant extract (RPMI without fetal bovine serum)

The cytotoxicity assay of the compound was expressed as EC_100_ and was calculated by the Graphpad Instat software using the % viability calculated from the serial dilutions of each plant extract.

## Detection of the effective anti-inflammatory concentrations (EAICs) of the used treatments in lipopolysaccharides (LPS)-stimulated human WBC's culture

**Reagents**

- Reference anti-inflammatory drugs: piroxicam serial dilution (3.125, 6.25, 12.5, 25 and 50μg/ml in culture media)
- MTT solution (5 mg/ml in PBS)
- Dimethylsulfoxide (DMSO)
- LPS (1mg /ml in culture media)
- Culture media (RPMI containing 10% fetal bovine and 2% L- glutamine)

**Procedure**

In a 96 well plat, A volume of 50 μl of the culture medium that contained 100,000 of human WBCs was dispensed per well. The inflammation was induced by adding 50 μl of LPS to the plated cells and incubated in CO_2_ incubator. After 24 h, the plate was centrifuged at 1650 rpm for 5 min and the supernatants were discarded and then 200 µl of serial concentrations (0, 3.125, 6.25, 12.5, 25 and 50μg/ml in culture media) of the crude extracts or the standard anti-inflammatory drugs piroxicam were added. The control cells contained cell culture medium only. The plates were incubated for additional 72 h in CO_2_ incubator. After 72 h of incubation, the cell proliferations were measured using MTT (as previously illustrated). Stimulation index (SI) was used to assess the cell proliferations.

**Stimulation index** = (mean absorbance of LPS-stimulated cells or LPS-stimulated cells treated with different concentrations of natural product / absorbance of control untreated cells).

The effective anti-inflammatory concentration (EAICs) of each extract that were able to bring back the abnormal proliferation of LPS-stimulated cells to normal proliferation of control untreated cells (SI = 1) were calculated using Instate graph pad.

## Extraction of RNA of untreated and treated LPS-stimulated human white blood cells

**Reagents**

- RNA extraction kit
- Working wash buffer (100 µl wash buffer + 300 µl absolute ethanol)

**Procedure**

Cell pellets were then suspended in 50 µl of solution R1, mixed thoroughly 30 s, incubated at room temperature for 1 min, added 300 µl of solution R2, then mixed thoroughly 30 s and centrifuged at 4ºC for 3-5 min. The supernatant was transferred into a spin column and centrifuged for 30 s at 14000 rpm at 4ºC. The flow-through was discarded and 300 µl of working wash buffer was added into the spin column, centrifuged for 30 s (this step was repeated twice). The spin column was recentrifuged for 1min at 10,000 rpm and transferred to a sterile 1.5 ml micro centrifuge tube. Thirty microliters of elution buffer were added to the central of the membrane, incubated at room temperature for 1 min and then centrifuged for 30 s at 14000 rpm at 4ºC. The optical density (OD) of the extracted RNA was determined by measuring the absorbance and purity at A260 and A260/A280 nm, respectively using spectrophotometer and kept in -80°C until real time PCR.

## cDNA synthesis from RNA extracted from untreated and treated LPS-stimulated human white blood cells

**Reagents**

- cDNA Extraction kit.
- Nuclease-free water (non template negative control).
- dT Primer.
- Reaction buffer 5X
- RNase inhibitor 20 U/µl
- dNTPs 10 mM
- Reverse transcriptase 200 U/µl

**Procedure**

In PCR tubes, two µg of total RNA or nuclease-free water and 1 µl of oligo dT primer were added to nuclease-free water in a total volume of 12 µl, mixed gently, centrifuged, incubated at 65ºC for 5 min in PCR machine and placed back on ice immediately. Four microliters of 5X reaction buffer, 1 µl of RNase inhibitor, 2 µl of dNTPs mix and 1 µl of reverse transcriptase or 1 µl of nuclease-free water instead of reverse transcriptase for reverse transcriptase negative control were mixed gently with previous mixture, spin down and incubated for 60 min at 42ºC followed by heat inactivation at 70ºC for 5 min in PCR machine.

##

## Determination of IL1B, IL 6, TNF, INF and GAPDH expression level by real time polymerase chain reaction (PCR)

**Reagents**

- SYBR green master mix 2X (containing hot start Thermus aquaticus (Taq) DNA Polymerase in an optimized buffer with a final concentration of 2.5 mM MgCl_2_, uracil DNA glycosylase (UDG), dNTPs and double-stranded DNA binding dye SYBR green).
- cDNA
- Nuclease-free water

**Procedure**

In PCR tubes, a volume of 12.5 µl of 2 X SYBR green master mix was mixed with 5µl of cDNA, 0.5 µl of 10 pmoles/ml forward primer and 0.5 µl of pmoles/ml reverse primer for each primer. As for the reference tube, 0.5 µl of 10 pmoles/ml forward primer of β-actin and 0.5 µl of 10 pmoles/ml for reverse primer of β- actin were added. The other tube was used as a non-template control (NTC), to assess for reagent contamination or primer dimers by adding 1 µl of nuclease-free water instead of template used. The tubes were gently mixed with 6.5 µl nuclease free water without creating bubbles (bubbles will interfere with the fluorescence detection) and spinned for few seconds. Samples were placed in the cycler and start the program as follows; 1 cycle of 95ºC for 10 min (initial denaturation), followed by 40 cycles of 95ºC for 15 sec (denaturation), 60ºC for 30s (annealing) and 72ºC for 30s (extension).

**Calculation**

**Expressions fold levels of gene calculated by**

ΔCt _normal_ =Ct _normal untreated cells_ – Ct _reference_

ΔCt _tested plant extract_ = Ct _tested plant extract-treated cells_ – Ct _reference_

ΔCt _induced_ =- Ct _LPS-exposed cells_ – Ct _reference_

**In case of genes:**

ΔΔCT _tested plant extract_ = ΔCt _tested plant extract_– ΔCt _normal_

ΔΔCT _induced_ = ΔCt _induced_ – ΔCt _normal_

**In case of GAPDH:**

ΔΔCT _tested plant extract_ = ΔCt _normal_ - ΔCt _tested plant extract_

ΔΔCT _induced_ = ΔCt _normal_ - ΔCt _induced_

**Fold change in gene expression = log (2^-ΔΔCT^)**

**Where:**

**Ct _tested plant extract_**: threshold cycle value of genes of extracted mRNA of plant extract treated-LPS-stimulated WBCs which is defined as the cycle number at which the fluorescence generated within a reaction crosses the fluorescence threshold.

**Ct _reference_**: threshold cycle value of GAPDH which is used for normalization.

**Ct _normal_**: threshold cycle value of genes of extracted mRNA of untreated control WBCs

**Ct _induced_**: threshold cycle value of gene of extracted mRNA of LPS-stimulated WBCs

Primers

| TNF alpha | F-CTCTTCTGCCTGCTGCACTTTG |
| --- | --- |
|  | R- ATGGGCTACAGGCTTGTCACTC |
| IL 6 | F, 5′-TGAACTCCTTCTCCACAAGCG-3′ |
|  | R, 5′-TCTGAAGAGGTGAGTGGCTGTC-3′ |
| IL 1B | F, CCACAGACCTTCCAGGAGAATG |
|  | R, GTGCAGTTCAGTGATCGTACAGG |
| INF | F, GAGTGTGGAGACCATCAAGGAAG |
|  | R, TGCTTTGCGTTGGACATTCAAGTC |
| GAPDH | F, GGATTTGGTCGTATTGGG |
|  | R, GGAAGATGGTGATGGGATT |

**Reference**

1. <http://www.sigmaaldrich.com/catalog/product/sigma/>mak081?lang=en&ion=JO.

2. Du, Y. *et al.* Activation of a caspase 3-related cysteine protease is required for glutamate-mediated apoptosis of cultured cerebellar granule neurons. *Proceedings of the National Academy of Sciences*. **94**(21), 11657-11662 (1997).

3. Louis, K. S., & Siegel, A. C. Cell viability analysis using trypan blue: manual and automated methods. *Methods in molecular biology (Clifton, N.J.)*, *740*, 7–12 (2011). https://doi.org/10.1007/978-1-61779-108-6_2
