# Supplementary material for: Forecasting of potential anti-inflammatory targets of some immunomodulatory plants and their constituents using in vitro, molecular docking and network pharmacology-based analysis
Source: Sci Rep. 2023 Jun 12;13:9539. doi: 10.1038/s41598-023-36540-3 (PMC10260966; doi:10.1038/s41598-023-36540-3)
Supplement: Supplementary file 5 — Supplementary Information 5. [file 41598_2023_36540_MOESM5_ESM.pptx]

## Slide 1
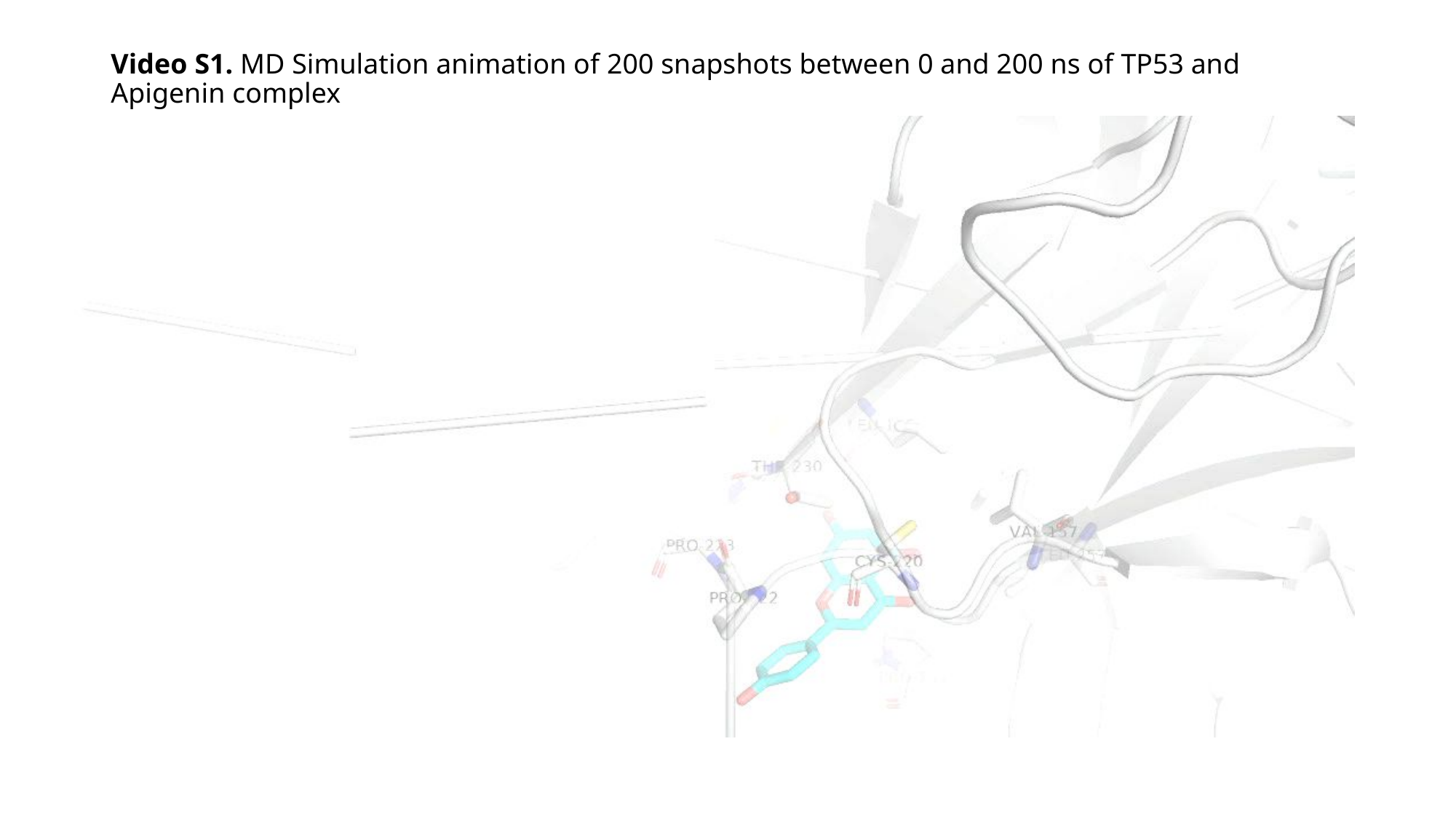

# Video S1. MD Simulation animation of 200 snapshots between 0 and 200 ns of TP53 and Apigenin complex

## Slide 2
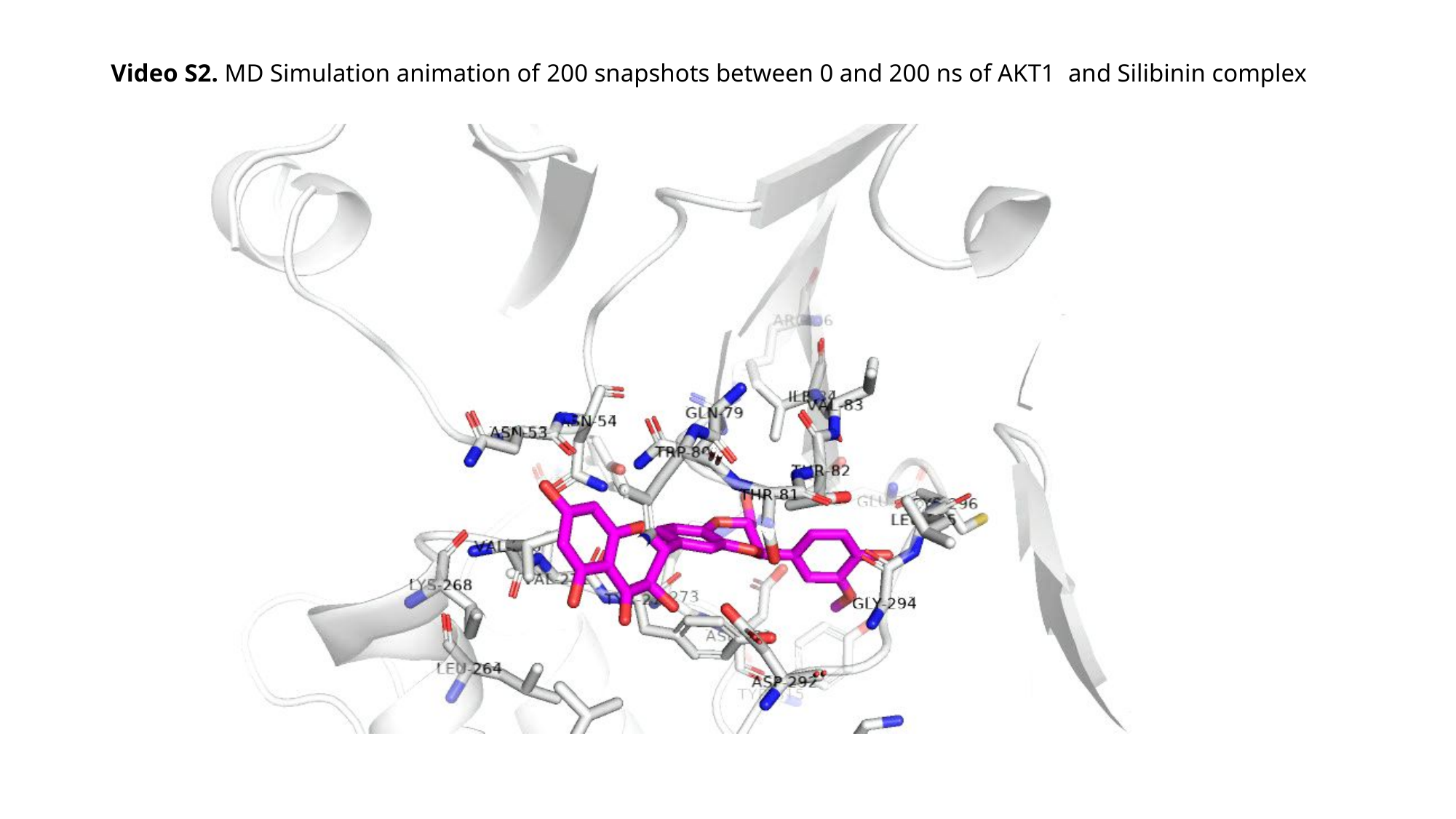

# Video S2. MD Simulation animation of 200 snapshots between 0 and 200 ns of AKT1 and Silibinin complex

## Slide 3
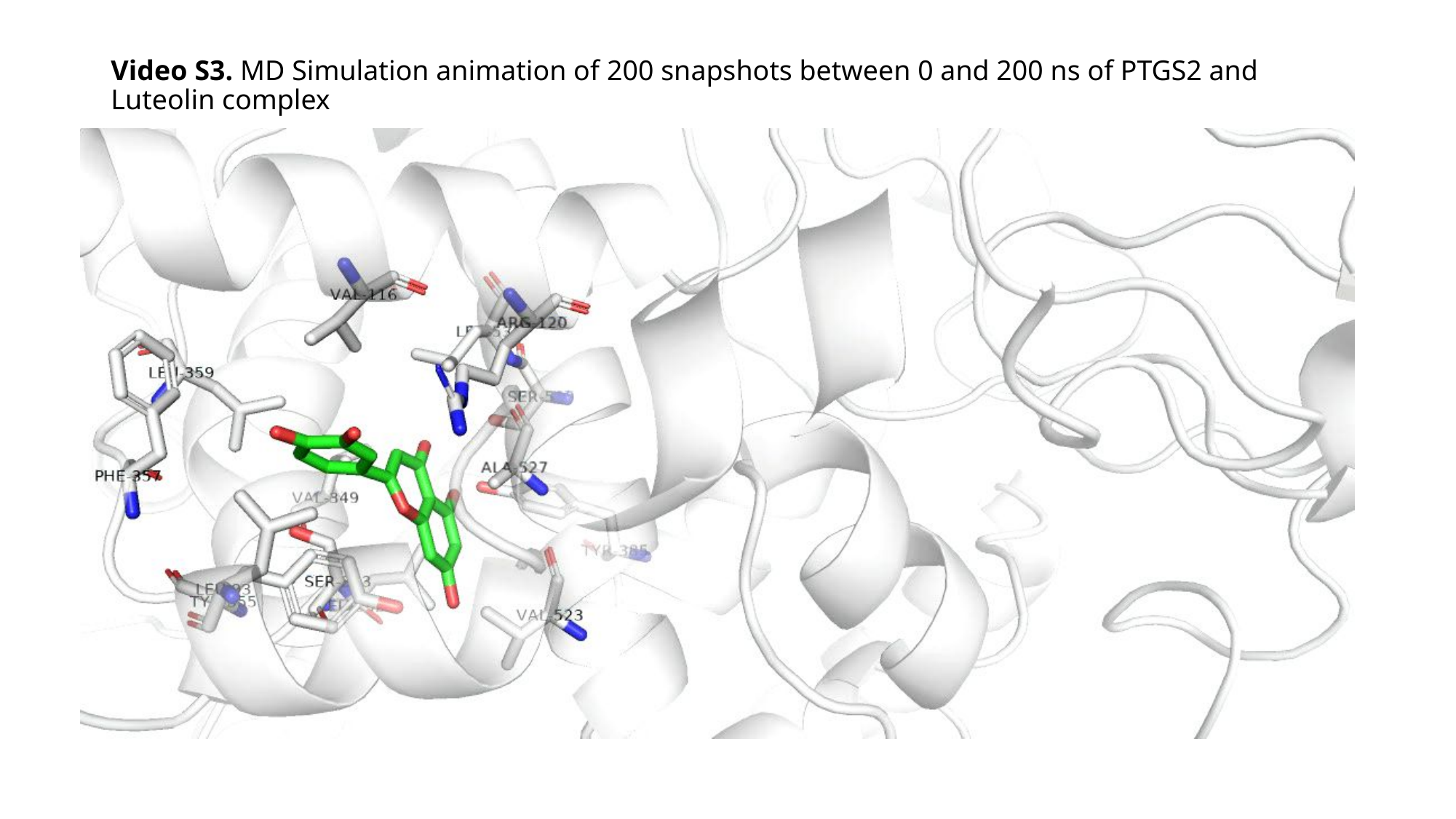

# Video S3. MD Simulation animation of 200 snapshots between 0 and 200 ns of PTGS2 and Luteolin complex
